# Strategic Decisions and the Entrepreneurial Mindset in Sustaining Agribusiness SMEs in Emerging Economies: Unlocking Pathways to Resilience – A Narrative Review

**DOI:** 10.12688/f1000research.177782.2

**Published:** 2026-05-04

**Authors:** Tom Ongesa Nyamboga

**Affiliations:** 1 bushesnyi, Kampala International University - Western Campus, Bushenyi, Western Region, Uganda

**Keywords:** Agribusiness SMEs, Strategic Decisions, Entrepreneurial Mindset, Sustainability, Emerging Economies

## Abstract

This narrative review examines how strategic decisions shaped by the entrepreneurial mindset sustain agribusiness SMEs in emerging economies, highlighting pathways to resilience. Recognising a critical knowledge gap in understanding the cognitive mechanisms that drive SME sustainability, the review is anchored in Entrepreneurial Cognition Theory and the Triple Bottom Line framework. The study synthesises empirical evidence from 2020 to 2025 using a qualitative research design with systematic data collection from peer-reviewed journals and grey literature. Findings indicate that entrepreneurial cognition guides strategic resource allocation, innovation adoption, market positioning, risk management, and the integration of social and environmental sustainability, collectively enhancing economic, social, and ecological resilience. Persistent challenges include structural constraints, unequal access to finance and technology, gender disparities, and sustainability trade-offs in niche markets. Policy and practical implications emphasise cooperative models, targeted innovation financing, market intelligence support, and incentives for sustainability-aligned practices. The review contributes empirically by consolidating evidence across Africa, Asia, and Latin America, offering comparative insights into how SMEs navigate complex and resource-constrained environments. Theoretically, it extends the application of Entrepreneurial Cognition Theory and the Triple Bottom Line framework to context-specific decision-making in agribusiness SMEs, demonstrating how cognitive processes underpin sustainable strategic actions. By linking entrepreneurial decision-making to resilience outcomes, the review provides actionable insights for policymakers, development partners, and SME owner-managers seeking to enhance competitiveness, long-term viability, and inclusive growth in emerging economies.

## Introduction

Sustainable agribusiness has become a critical pillar for economic resilience, food security, and inclusive growth in emerging economies. ^1^ Within these contexts, agribusiness small and medium-sized enterprises play a central role by anchoring agricultural value chains, linking smallholder production to markets, and generating employment across rural and peri-urban areas. ^2^ The sustainability of these enterprises extends beyond short-term profitability to encompass economic continuity, environmental stewardship, and social viability across interconnected production, processing, and distribution systems. ^3^ Rapid climate variability, fluctuating market conditions, institutional weaknesses, and persistent resource constraints increasingly shape the operating environment of agribusiness SMEs, intensifying the need for deliberate and forward-looking managerial action. ^4, 5^

Strategic decision-making represents a core mechanism through which agribusiness SMEs navigate this complexity. ^6, 7^ Decisions related to resource allocation, market positioning, innovation adoption, and stakeholder engagement determine how enterprises respond to uncertainty and build resilience over time. ^8^ In emerging economies, owner-managers often make such decisions under conditions of imperfect information, limited access to finance, and weak institutional support. ^9^ The entrepreneurial mindset plays a decisive role in this process by shaping how opportunities are identified, risks are assessed, learning is internalised, and long-term objectives are prioritised. ^10, 11^ Entrepreneurial cognition therefore influences not only the content of strategic decisions but also the manner in which sustainability-oriented strategies are conceived and implemented. Understanding the interaction between strategic decisions and entrepreneurial thinking is essential for explaining how agribusiness SMEs sustain performance in challenging environments.

Existing scholarship on agribusiness SMEs in emerging economies remains fragmented. Many studies focus on sustainability practices, entrepreneurial orientation, or firm performance as separate constructs, often without sufficient attention to the cognitive foundations that link entrepreneurial thinking to strategic decision-making. ^12, 13^ Research on strategic management tends to emphasise outcomes, while entrepreneurship literature frequently prioritises traits or orientations, leaving limited insight into how entrepreneurial mindsets actively shape strategic choices that support long-term sustainability. ^14, 15^ This separation restricts theoretical integration and weakens the practical relevance of findings for owner-managers and policymakers seeking to strengthen agribusiness resilience. The absence of a coherent synthesis is particularly problematic in emerging economies, where agribusiness SMEs operate within highly dynamic and constrained contexts. Without an integrated understanding of how strategic decisions emerge from entrepreneurial cognition and translate into sustained economic, environmental, and social outcomes, guidance for supporting these enterprises remains incomplete.

This study responds to this gap by critically synthesising interdisciplinary evidence through a narrative review approach, integrating insights from strategic management, entrepreneurship, and sustainability scholarship. The study examines how strategic decisions, shaped by the entrepreneurial mindset, contribute to the sustainability of agribusiness small and medium-sized enterprises in emerging economies. Beyond advancing conceptual clarity and contributing to the extant literature, the findings are intended to provide practical value to entrepreneurs and owner-managers by offering insight into how cognitive processes can be leveraged to improve decision-making under uncertainty, optimise resource allocation, and enhance adaptive capacity in volatile markets. The synthesis also offers guidance on aligning business strategies with sustainability goals, enabling entrepreneurs to strengthen long-term viability while responding to environmental and social expectations. In addition, the study provides policy-relevant insights by identifying strategic and cognitive factors that can inform capacity-building initiatives, financial support mechanisms, and institutional frameworks aimed at improving the resilience and competitiveness of agribusiness SMEs in emerging economies.

### Specific objectives

• To examine the nature and characteristics of strategic decisions made by agribusiness SMEs in emerging economies within contexts of uncertainty and resource constraints.
• To analyse the dimensions of the entrepreneurial mindset that influence strategic decision-making among owner-managers of agribusiness SMEs.
• To assess how entrepreneurial cognition shapes sustainability-oriented strategic choices across economic, environmental, and social dimensions.
• To evaluate the contribution of strategic decisions informed by an entrepreneurial mindset to the long-term performance and resilience of agribusiness SMEs.
• To identify gaps and emerging directions in existing literature that can inform integrated frameworks and policy interventions for sustaining agribusiness SMEs in emerging economies.

Guided by these objectives, the study addresses the central question: how do strategic decisions, shaped by the entrepreneurial mindset, influence the sustainability of agribusiness small and medium-sized enterprises in emerging economies?

## Materials and methods

### Data collection methods

The study employed a narrative review approach to synthesise literature on the role of entrepreneurial cognition in sustaining agribusiness SMEs in emerging economies. This approach was considered appropriate because it allows for flexible integration of diverse empirical, conceptual, and theoretical evidence, which is essential for examining complex and evolving phenomena such as entrepreneurial cognition and sustainability. Data were collected from peer-reviewed journal articles, working papers, and high-impact reports published between 2019 and 2025 as shown in Table 1. The selection of the 2019–2025 period was deliberately informed by the need to capture both pre-pandemic baseline evidence and post-pandemic transformations in agribusiness systems, entrepreneurial decision-making, and sustainability practices. This period reflects significant global and regional disruptions, including COVID-19, which reshaped SME resilience strategies, innovation adoption, and market dynamics, making it particularly relevant for this review.

**Table 1.** Methodological approach for the narrative review on entrepreneurial cognition and sustainability in agribusiness SMEs.

Methodological component	Details
**Data Collection Methods**	Systematic narrative review of peer-reviewed articles, working papers, and high-impact reports (2020–2025); databases included Scopus, Web of Science, Google Scholar, PubMed. Focused on agribusiness SMEs, entrepreneurial cognition, and sustainability outcomes.
**Search Keywords**	“Entrepreneurial cognition” AND “agribusiness SMEs”; “Resource allocation” OR “resource optimisation” AND “sustainability”; “Innovation adoption” OR “technological advancement” AND “emerging economies”; “Market positioning” OR “competitive advantage” AND “small enterprises”; “Risk management” OR “resilience” AND “agribusiness”; “Social sustainability” OR “environmental sustainability” AND “SMEs”. Boolean operators used to refine search.
**Screening and Inclusion Criteria**	Articles published 2020–2025; empirical, theoretical, or conceptual studies on agribusiness SMEs in emerging economies; focus on entrepreneurial cognition and sustainability; peer-reviewed or credible reports; English language.
**Exclusion Criteria**	Studies on large enterprises or non-agribusiness sectors; published before 2020; non-credible or insufficiently detailed sources; duplicates; inaccessible full text.
**Data Analysis**	Thematic synthesis mapping key information (country, SME type, cognitive factors, strategic decisions, sustainability outcomes) against Entrepreneurial Cognition Theory and Triple Bottom Line framework; comparative analysis across sub-Saharan Africa, South Asia, and Latin America.
**Evaluation and Rigour Enhancement**	Dual independent screening and extraction; credibility assessed via journal impact, methodology, and institutional affiliation; triangulation using empirical studies, conceptual papers, and reports; audit trails and memoing for transparency.
**Reflexivity**	Reflexive awareness of researcher perspectives and prior knowledge; monitored potential biases in study selection and interpretation; documented thematic decision-making, particularly regarding gender, informal SMEs, and contextual gaps.

Academic databases consulted included Scopus, Web of Science, Google Scholar, and PubMed, complemented by grey literature from dissertations. These databases were selected based on their credibility, interdisciplinary scope, and relevance to entrepreneurship, sustainability, and agricultural research. Scopus and Web of Science ensured access to high-quality, peer-reviewed and indexed studies, Google Scholar broadened the search to include emerging and cross-disciplinary contributions, while PubMed supported access to studies linking agribusiness with environmental and public health dimensions. The collection process emphasised studies providing empirical, conceptual, or theoretical insights on entrepreneurial resource allocation, innovation adoption, market positioning, risk management, and social and environmental sustainability in agribusiness contexts.

### Search keywords

Searches employed a combination of Boolean operators to capture relevant literature. Core keywords included:

• “Entrepreneurial cognition” AND “agribusiness SMEs”
• “Resource allocation” OR “resource optimisation” AND “sustainability”
• “Innovation adoption” OR “technological advancement” AND “emerging economies”
• “Market positioning” OR “competitive advantage” AND “small enterprises”
• “Risk management” OR “resilience” AND “agribusiness”
• “Social sustainability” OR “environmental sustainability” AND “SMEs”

These terms were adapted and combined to ensure comprehensive coverage across economic, social, and environmental dimensions of SME sustainability. The selection and refinement of keywords reflected contemporary terminology and evolving research priorities within the 2019–2025 period.

### Screening and inclusion criteria

The initial search yielded over 1,200 articles, which were screened in multiple stages. Inclusion criteria comprised:

• Studies published between 2019 and 2025.
• Empirical, conceptual, or theoretical works focusing on agribusiness SMEs in emerging economies.
• Articles addressing at least one aspect of entrepreneurial cognition, resource allocation, innovation, market positioning, risk management, or sustainability outcomes.
• Peer-reviewed journal articles, high-impact reports, and credible working papers in English.

The inclusion of studies from 2019 ensured that foundational pre-pandemic insights were captured, while extending to 2025 allowed incorporation of the most recent evidence on resilience, recovery, and sustainability transitions. This range is particularly appropriate for a narrative review, which seeks to provide a comprehensive and contextually rich synthesis rather than a narrowly bounded systematic assessment.

### Exclusion criteria

Studies were excluded if they:

• Focused on large enterprises or non-agribusiness sectors.
• WWere published before 2019 or in non-peer-reviewed sources without credibility.
• Lacked sufficient methodological detail, empirical evidence, or relevance to sustainability outcomes.
• Were duplicates or inaccessible in full-text format.

This exclusion approach ensured alignment with the defined time frame while maintaining relevance, credibility, and coherence in the body of evidence reviewed.

### Data analysis

A thematic synthesis approach guided data analysis. Key information extracted from each study included country context, SME type, entrepreneurial cognitive factors, sustainability outcomes, strategic decisions, and policy implications. Themes were mapped against the conceptual framework combining Entrepreneurial Cognition Theory and the Triple Bottom Line framework to identify patterns, contradictions, and research gaps. Comparative analysis across sub-Saharan Africa, South Asia, and Latin America was conducted to highlight contextual variations and generalisable insights within the 2019–2025 evidence base, capturing both pre- and post-pandemic dynamics.

### Evaluation and rigour enhancement

To enhance rigour within the narrative review design, several strategies were applied. Two independent reviewers conducted screening, extraction, and coding of studies, resolving discrepancies through discussion. Source credibility was evaluated based on journal impact, institutional affiliation, and methodological transparency. Data triangulation was achieved by integrating empirical studies, conceptual articles, and policy reports. The use of established databases such as Scopus and Web of Science strengthened the reliability of included sources, while Google Scholar and grey literature enhanced the breadth of coverage. Memoing and audit trails documented the analytical process, supporting transparency and consistency without imposing the rigid protocols associated with systematic reviews.

### Reflexivity

The review maintained reflexivity by recognising the influence of the researchers’ perspectives and prior knowledge of agribusiness SMEs. Attention was paid to potential biases in study selection, interpretation, and thematic synthesis, particularly in relation to the chosen time frame and database coverage. Reflexive notes guided the identification of dominant narratives and gaps, especially in underrepresented contexts such as gendered access to resources and informal SME structures. This approach ensured that the narrative synthesis critically engaged with both empirical evidence and theoretical assumptions within the 2019–2025 scope without introducing unintentional bias.

### Data collection methods

The study employed a systematic narrative review approach to synthesise literature on the role of entrepreneurial cognition in sustaining agribusiness SMEs in emerging economies. Data were collected from peer-reviewed journal articles, working papers, and high-impact reports published between 2019 and 2025 as shown in Table 1. Academic databases consulted included Scopus, Web of Science, Google Scholar, and PubMed, complemented by grey literature from dissertations. The collection process emphasised studies providing empirical, conceptual, or theoretical insights on entrepreneurial resource allocation, innovation adoption, market positioning, risk management, and social and environmental sustainability in agribusiness contexts.

### Theoretical framework

The review is anchored on Entrepreneurial Cognition Theory as articulated by Mitchell et al. in 2002 as shown in Table 2. The theory posits that entrepreneurs differ from non-entrepreneurs in the way information is perceived, processed, and interpreted, particularly under conditions of uncertainty and constraint. ^16^ Entrepreneurial cognition emphasises mental models, scripts, and heuristics that guide opportunity recognition, risk assessment, learning, and strategic judgement. ^17^ The core assumption of the theory is that strategic decisions do not arise solely from objective analysis of external conditions but are shaped by the cognitive frameworks of decision-makers, which influence how opportunities and threats are recognised and acted upon. ^18^ Within agribusiness SMEs in emerging economies, owner-managers operate in volatile environments characterised by climate shocks, institutional gaps, and market imperfections. Entrepreneurial cognition explains how such actors make strategic decisions despite limited information and resources. In this study, the theory is significant because it explains how the entrepreneurial mindset shapes strategic decision-making processes that ultimately influence sustainability outcomes. It provides a lens for understanding why agribusiness SMEs facing similar constraints may adopt divergent strategies with different long-term implications.

**Table 2.** Theoretical framework linking entrepreneurial cognition, strategic decisions, and sustainability outcomes in agribusiness SMEs.

Framework component	Key theoretical focus	Core explanation within the study
Entrepreneurial cognition independent variable	Mental models, scripts, heuristics, opportunity recognition, and risk perception	Explains how owner-managers perceive, interpret, and act on information under uncertainty, shaping strategic judgement in resource-constrained agribusiness environments
Strategic decision-making	Resource allocation, innovation adoption, market positioning, risk management	Represents the strategic choices influenced by entrepreneurial mindset that translate cognition into observable organisational actions
Sustainability outcomes	Economic viability, environmental stewardship, social responsibility	Captures firm-level sustainability as a multidimensional outcome reflecting long-term financial performance, ecological protection, and social inclusion
Theoretical anchor I	Entrepreneurial Cognition Theory	Provides the lens for understanding why firms facing similar external conditions adopt different strategies with varying long-term implications
Theoretical anchor II	Triple Bottom Line Theory	Offers a structured basis for evaluating sustainability by integrating economic, environmental, and social dimensions

The review is also anchored on the Triple Bottom Line Theory proposed by Elkington in 1997 to explain sustainability. The theory posits that organisational sustainability extends beyond financial performance to include environmental stewardship and social responsibility, arguing that long-term enterprise survival depends on balancing these three interrelated dimensions. ^19^ The underlying assumption is that firms operating sustainably create value not only for owners but also for society and the natural environment, thereby enhancing resilience and legitimacy over time. In the context of agribusiness SMEs, the theory explains sustainability as a multidimensional outcome shaped by strategic choices related to production practices, resource use, market engagement, and social inclusion. The Triple Bottom Line framework is particularly relevant to emerging economies, where agribusiness activities directly affect food security, rural livelihoods, and ecological systems. ^20^ Within this study, the theory provides a structured basis for assessing sustainability outcomes and highlights why strategic decisions influenced by entrepreneurial thinking must align economic viability with environmental and social considerations. Together, these theories offer an integrated foundation for examining how entrepreneurial cognition informs strategic decisions that sustain agribusiness SMEs over time.

### Literature review

How strategic decisions, shaped by the entrepreneurial mindset, influence the sustainability of agribusiness small and medium-sized enterprises in emerging economies as shown in Table 3.

**Table 3.** Strategic decision areas shaped by the entrepreneurial mindset and their influence on the sustainability of agribusiness SMEs in emerging economies.

Major strategic area	Entrepreneurial decision focus	Key sustainability outcomes
Resource allocation and optimisation	Prioritising high-impact investments, flexible deployment of capital and labour, and use of locally available inputs	Improved productivity, cost efficiency, resilience to shocks, strengthened local economies, reduced environmental footprint
Innovation adoption and technological advancement	Adoption of process, product, and digital innovations aligned with resource constraints and market opportunities	Enhanced efficiency, reduced post-harvest losses, expanded market access, improved environmental performance
Market positioning and competitive advantage	Targeting niche markets, brand differentiation, adaptive pricing, and value chain integration	Stable revenues, premium pricing, stronger competitiveness, social inclusion through ethical and fair-trade practices
Risk management and resilience building	Diversification, proactive environmental and financial risk strategies, contingency planning	Business continuity, reduced vulnerability to climate and market shocks, long-term economic stability
Social and environmental sustainability integration	Embedding community development, responsible resource use, and regulatory compliance into strategy	Social equity, environmental conservation, reputational strength, long-term viability
Social and environmental sustainability integration	Embedding community development, responsible resource use, and regulatory compliance into strategy	Social equity, environmental conservation, reputational strength, long-term viability

### Resource allocation and optimisation

Resource allocation represents a critical area where the entrepreneurial mindset directly shapes the sustainability of agribusiness SMEs in emerging economies. ^21^ Entrepreneurial owner-managers often face severe constraints in financial, human, and material resources, requiring strategic prioritisation of investments. ^22^ By identifying high-impact areas that promise maximum returns, entrepreneurs ensure that scarce resources are channelled into initiatives that enhance productivity, market competitiveness, and long-term viability. ^23^ For instance, in Kenya, small-scale horticulture enterprises strategically invest in drip irrigation systems rather than broad-scale mechanised farming, optimising limited water resources while increasing yields for export markets. ^24^ Similarly, in Bangladesh, aquaculture SMEs prioritise investment in high-quality fingerlings and feed, which maximises production efficiency and supports the firm’s profitability within resource-constrained rural settings. ^25, 26^

Entrepreneurial thinking also drives cost-efficient operations, fostering lean management practices that reduce waste and improve overall sustainability. ^27, 28^ In Nigeria, cassava processing SMEs adopt modular production techniques that minimise raw material loss while enabling flexible responses to fluctuating market demand. ^29, 30^ In Ethiopia, small coffee cooperatives implement shared roasting and storage facilities, lowering operational costs for individual farmers and supporting collective economic resilience. ^31^ Such approaches illustrate how strategic decisions guided by entrepreneurial cognition enable SMEs to maintain financial stability while optimising production processes.

Flexibility in resource deployment is another significant dimension. SMEs with an entrepreneurial orientation can quickly reallocate capital, labour, or equipment in response to environmental shocks or sudden market shifts. ^32^ In Ghana, small-scale cocoa processors shift resources between local and international markets depending on price fluctuations and regulatory changes, ensuring business continuity. ^33^ In Uganda, poultry SMEs adjust feed allocation and flock size in response to seasonal disease outbreaks, balancing operational sustainability with economic returns. ^34^ This adaptive capacity highlights the critical role of entrepreneurial cognition in supporting resilience amidst uncertainty.

Strategic integration of locally available inputs strengthens both environmental and social sustainability. ^35, 36^ By sourcing inputs within the immediate community, agribusiness SMEs reduce dependency on distant supply chains, lower transport-related emissions, and contribute to local economic development. ^37^ In India, small vegetable farming enterprises favour indigenous seed varieties and composted organic fertilisers, enhancing soil health while reducing production costs. ^38^ In Tanzania, small dairy processors rely on locally sourced fodder and veterinary services, which supports surrounding communities and ensures the continuity of supply chains. ^39^ Through such decisions, resource allocation becomes not only a matter of efficiency but also a mechanism for embedding sustainability within the operational fabric of agribusiness SMEs in emerging economies.

### Innovation adoption and technological advancement

Innovation adoption is a critical pathway through which the entrepreneurial mindset influences the sustainability of agribusiness SMEs in emerging economies. ^21, 40^ Entrepreneurs with a strong cognitive orientation towards opportunity recognition and problem-solving are more likely to embrace innovative practices that enhance productivity, efficiency, and competitiveness. ^41^ In Vietnam, for example, small rice-processing SMEs have adopted mechanised milling technologies, which improve grain quality, reduce post-harvest losses, and enable access to both domestic and export markets. ^42^ This technological integration demonstrates how strategic decisions informed by entrepreneurial thinking can simultaneously support economic sustainability and reduce environmental waste. ^43^

Process innovation is another dimension where entrepreneurial cognition drives sustainability-oriented outcomes. ^44^ In Kenya, small-scale dairy cooperatives have implemented automated milk pasteurisation and cooling systems, enabling farmers to extend product shelf life while maintaining hygiene standards. ^45^ This approach not only improves profitability but also enhances social sustainability by ensuring safe, nutritious products for local communities. Similarly, in Peru, quinoa-processing SMEs have introduced solar drying techniques to replace firewood-based methods, reducing carbon emissions while preserving product quality. ^46, 47^ Such process-level innovations exemplify the environmental and economic benefits that arise from entrepreneurial-driven strategic choices.

Entrepreneurial decision-making also supports the adoption of digital and market-based technologies, which expand market reach and improve operational efficiency. ^48, 49^ In India, small mango and banana exporters utilise mobile-based platforms to monitor prices, forecast demand, and connect with buyers across regional and international markets. ^50^ In Nigeria, cassava-processing SMEs use simple smartphone applications to track inventory and manage supply chain logistics, ensuring timely delivery and reducing post-harvest losses. ^51^ These technological innovations are strategic decisions informed by the owner-managers’ cognitive ability to recognise opportunities and mitigate market risks, directly contributing to business continuity.

Sustainability-focused innovations are often prioritised in agribusinesses with an entrepreneurial mindset. ^52^ In South Africa, small olive oil producers have integrated drip irrigation and organic fertilisers to optimise water usage and maintain soil fertility, reflecting a conscious alignment of economic and environmental objectives. ^53^ In Bangladesh, shrimp aquaculture SMEs employ recirculating water systems that minimise water consumption and reduce pollution, balancing profitability with environmental stewardship. ^25^ These examples demonstrate how entrepreneurial cognition guides strategic decisions that embed innovation as a mechanism for achieving the triple bottom line of economic, environmental, and social sustainability in agribusiness SMEs across diverse emerging economies.

### Market positioning and competitive advantage

Market positioning represents a strategic domain where the entrepreneurial mindset significantly influences the sustainability of agribusiness SMEs in emerging economies. ^54^ Entrepreneurs with strong opportunity recognition and risk-assessment capabilities strategically identify profitable and underserved market segments, enabling their enterprises to gain competitive advantage. ^55^ In Colombia, small coffee cooperatives have positioned themselves in niche fair-trade and organic markets, attracting premium prices while promoting environmentally responsible practices. ^56^ Similarly, in Indonesia, small palm sugar producers have targeted the health-conscious segment by branding products as natural and artisanal, differentiating themselves from mass-produced competitors. ^57^ These strategic decisions demonstrate how entrepreneurial cognition informs market-focused choices that enhance both economic and social sustainability.

Brand differentiation also plays a central role in sustaining agribusiness SMEs. ^58, 59^ In Morocco, argan oil cooperatives have developed strong local and international brand identities emphasising quality, ethical sourcing, and women’s empowerment. ^60^ This positioning not only allows higher market prices but also strengthens social sustainability by supporting community livelihoods. ^61^ In the Philippines, small cacao processors have distinguished their products through origin-based marketing, highlighting traceability and ethical production practices. ^62^ Such strategic decisions reflect the entrepreneurial mindset in action, linking innovative market approaches to long-term sustainability.

Entrepreneurial cognition further informs adaptive pricing strategies that balance profitability with market accessibility. ^62, 63^ In Egypt, small vegetable farmers adjust pricing seasonally to respond to market supply fluctuations, protecting both income and customer base. ^64^ In Guatemala, small-scale maize millers adopt flexible pricing structures for local and regional buyers, ensuring competitiveness while maintaining steady cash flow. ^65, 66^ These decisions exemplify how strategic responsiveness to market conditions, guided by entrepreneurial thinking, supports both economic continuity and market resilience.

Strategic integration within the value chain strengthens competitiveness and operational sustainability. ^67, 68^ In Bolivia, quinoa cooperatives coordinate with smallholder farmers, processors, and exporters to ensure consistent quality and supply, enhancing both market reliability and economic returns. ^69^ In Sri Lanka, cinnamon SMEs establish direct links with international buyers, reducing intermediary costs and improving profitability for local producers. ^70^ By making deliberate decisions that optimise market positioning and value chain integration, agribusiness SMEs leverage their entrepreneurial mindset to secure competitive advantage while achieving economic, social, and environmental sustainability.

### Risk management and resilience building

Risk management and resilience represent crucial areas where the entrepreneurial mindset shapes strategic decisions to sustain agribusiness SMEs in emerging economies. ^71, 72^ Entrepreneurs with strong cognitive orientation anticipate market volatility, environmental shocks, and institutional uncertainties, allowing them to adopt proactive strategies that safeguard both economic and operational stability. ^73^ In Zimbabwe, small maize milling SMEs diversify their sources of raw maize and storage facilities to mitigate the effects of seasonal crop failures and fluctuating prices, ensuring continuity of supply and business survival. ^74^ In Cambodia, small shrimp aquaculture enterprises employ early warning systems for water quality and disease outbreaks, allowing timely interventions that minimise losses and maintain productivity. ^75^

Strategic diversification of products and services is another key outcome of entrepreneurial thinking. ^76^ In Senegal, small groundnut-processing SMEs expand into producing peanut oil and packaged snacks alongside raw peanuts, reducing dependence on a single revenue stream while enhancing market reach. ^77^ In Nepal, small-scale vegetable farms rotate crops between leafy greens, root vegetables, and fruits, balancing income sources and reducing vulnerability to pest infestations or market price fluctuations. ^78^ Such decisions demonstrate how entrepreneurial cognition translates into deliberate actions that enhance resilience and long-term economic sustainability.

Financial risk mitigation is also central to sustainability. ^79, 80^ In Peru, small cacao cooperatives establish collective savings and microcredit schemes to buffer against market shocks and input shortages, ensuring steady operations. ^81^ In Vietnam, small aquaponics SMEs strategically reinvest profits into emergency funds and backup infrastructure, enabling rapid recovery from floods or supply chain disruptions. ^82^ These financial strategies, guided by entrepreneurial mindset, illustrate the link between strategic decision-making and sustained firm performance under uncertainty.

Environmental risk adaptation underscores the integration of resilience and sustainability. ^83, 84^ In Kenya, small tea farmers implement terracing and agroforestry practices to prevent soil erosion and mitigate the impact of erratic rainfall. ^85^ In Bolivia, quinoa producers adopt frost-resistant varieties and water-conserving irrigation techniques to protect yields from climate variability. ^86^ By embedding environmental risk management into strategic choices, agribusiness SMEs not only safeguard production but also promote long-term ecological sustainability. These examples highlight how entrepreneurial cognition drives strategic decisions that simultaneously address economic, environmental, and social risks, enhancing the resilience of agribusiness SMEs in diverse emerging economies.

### Social and environmental sustainability integration

Integrating social and environmental considerations into strategic decisions is a key pathway through which the entrepreneurial mindset sustains agribusiness SMEs in emerging economies. ^21, 87^ Entrepreneurs with strong opportunity recognition and long-term vision deliberately embed practices that benefit communities and ecosystems while maintaining profitability. ^88^ In Malawi, small soybean-processing SMEs prioritise employing local youth and women, fostering social inclusion while strengthening local supply chains. This approach simultaneously enhances community livelihoods and builds a loyal workforce, which contributes to the firm’s long-term operational stability. ^89^ In Indonesia, small organic rice farmers implement cooperative models that ensure fair profit-sharing among members, supporting equitable development and social cohesion. ^90^

Responsible resource use reflects another dimension of sustainability integration. ^91^ In Egypt, small-scale date farms adopt solar-powered water pumps and drip irrigation systems to optimise water use in arid environments, reducing environmental stress while maintaining crop productivity. ^92^ In Colombia, small cacao farms practice shade-grown cultivation methods that preserve local biodiversity, demonstrating how strategic decisions informed by entrepreneurial thinking can align economic goals with ecological responsibility. ^93^ These practices illustrate the importance of sustainability-oriented cognition in guiding decisions that protect natural resources while supporting business continuity.

Compliance with environmental and social regulations is also shaped by entrepreneurial cognition. ^94, 95^ In the Philippines, small fishery SMEs adopt traceability systems and adhere to local certification requirements, ensuring market access for premium domestic and export markets. ^96^ In Tanzania, small vegetable farmers comply with organic certification standards, allowing them to access international markets and attract environmentally conscious consumers. ^97^ By strategically aligning operations with regulatory frameworks, SMEs mitigate legal and reputational risks while reinforcing sustainability outcomes. ^98^

The long-term vision embedded in the entrepreneurial mindset ensures that social and environmental objectives are integrated into strategic planning. ^99, 100^ In Morocco, small argan oil cooperatives invest in community education programs on sustainable harvesting practices, protecting natural resources and promoting social development. ^101^ In Bangladesh, small shrimp farmers implement mangrove-friendly aquaculture practices, balancing environmental protection with economic returns. ^102^ These examples illustrate how entrepreneurial cognition guides strategic decisions that simultaneously advance social equity, environmental stewardship, and economic sustainability, ensuring that agribusiness SMEs in emerging economies remain resilient and viable over the long term.

## Discussion of literature

The literature review is analyzed to identify similar findings, contradictions, existing gaps and areas requiring further research. Again, a comparative evaluation with previous studies is done to highlight similarities and explain any differences observed in the findings. Finally, policy interventions are identified as shown in Table 4.

**Table 4.** Summary of major findings, gaps, comparative insights, and policy interventions linking entrepreneurial mindset to agribusiness SME sustainability.

Independent Variable (IV)	Key findings	Contradictions/Gaps	Comparative evaluation	Policy interventions
**Entrepreneurial Resource Allocation**	Strategic prioritisation of scarce financial, human, and material resources enhances productivity, cost-efficiency, resilience, and long-term sustainability ^21– 31^	Limited attention to inclusiveness, gender, power dynamics, and environmental consequences of intensification ^28, 31^	Local input integration and flexible deployment improve resilience across SSA and South Asia; contextual variations in market and institutional capacity affect outcomes ^32– 39^	Support cooperative/shared-resource models, expand SME training, provide access to finance and risk-mitigation instruments ^21, 31, 39^
**Innovation Adoption & Technological Advancement**	Adoption of process, product, and digital innovations enhances productivity, market access, and environmental stewardship ^21, 25, 40– 54^	Barriers of cost, skills, and infrastructure; uneven adoption among smaller or remote SMEs; long-term social impacts underexplored ^41, 49^	Sustainability-focused and digital innovations enhance resilience across Africa, South Asia, and Latin America; differences due to policy and infrastructure gaps ^25, 46, 50^	Innovation financing, technical training, investment in rural digital/energy infrastructure, incentives for sustainable technologies ^21, 48, 53^
**Market Positioning & Competitive Advantage**	Niche targeting, brand differentiation, adaptive pricing, and value chain integration improve economic returns and social value creation ^54– 70^	Durability of niche positioning, certification costs, and power asymmetries limit inclusivity and long-term competitiveness ^59, 68^	Adaptive pricing and direct buyer links enhance resilience; contextual differences in market access and institutional support ^63, 64, 70^	Trade facilitation, branding/certification support, inclusive value chain governance, entrepreneurship training ^54, 67^
**Risk Management & Resilience**	Proactive risk anticipation, diversification, financial buffering, and environmental adaptation strengthen continuity and sustainability ^71– 86^	Limited capacity of smaller SMEs to adopt sophisticated tools; gaps in social capital, informal risk-sharing, gender dynamics, and longitudinal evidence ^80, 83^	Integrated economic, financial, and environmental strategies reduce vulnerability across Africa, Asia, Latin America; differences reflect climate exposure and institutional support ^77, 81, 85^	Expand access to insurance, early warning systems, climate information, cooperative finance, and resilience-focused extension support ^71, 79, 84^
**Social & Environmental Sustainability Integration**	Inclusive employment, equitable value distribution, responsible resource use, and regulatory compliance enhance social and ecological outcomes alongside profitability ^87– 102^	High costs of adoption, feasibility constraints, informal enterprise inclusion, long-term trade-offs between profitability and environmental protection underexplored ^94, 95^	Traceability, certification, and community-centred approaches improve sustainability; context-specific variations due to regulatory, cultural, and ecological differences ^97, 101, 102^	Subsidies and technical support for compliance, cooperative governance, green finance access, incentives for inclusive employment and ecosystem conservation ^87, 95, 100^

### Entrepreneurial resource allocation and agribusiness SME sustainability

The literature demonstrates strong convergence on the positive relationship between entrepreneurial resource allocation and the sustainability performance of agribusiness SMEs in emerging economies, establishing a clear linkage between the independent variable of entrepreneurial mindset–driven allocation decisions and the dependent variable of long-term economic, social and environmental sustainability. ^21– 23^ Studies consistently show that strategic prioritisation of scarce financial, human and material resources enhances productivity, operational efficiency and resilience, as illustrated in horticulture enterprises in Kenya, aquaculture SMEs in Bangladesh, and processing firms in Nigeria and Ethiopia. ^24– 31^ These findings mirror earlier research that associates entrepreneurial orientation with lean management, cost minimisation and adaptive capacity in volatile agrarian contexts. ^22, 27, 28^ However, contradictions emerge regarding the inclusiveness and scalability of such optimisation strategies. While efficiency gains and profitability improvements are well documented, limited empirical attention has been paid to how these practices affect labour intensity, smallholder inclusion and intra-community equity over time. Some prior studies caution that resource optimisation may favour relatively better-capitalised SMEs, potentially excluding more vulnerable actors, an issue not fully explored in the reviewed cases. ^28, 31^ Noticeable gaps also exist around the influence of gender, informal norms and power relations on resource allocation decisions, alongside insufficient longitudinal evidence on the environmental consequences of sustained intensification. These gaps indicate the need for deeper, context-sensitive research examining how entrepreneurial resource strategies shape sustainability trajectories beyond short-term performance indicators.

Comparative evaluation with earlier studies confirms that the integration of locally available inputs and flexible resource deployment strengthens the pathway from entrepreneurial decision-making to sustainability outcomes, reinforcing both economic viability and community-level benefits. ^35– 39^ Similar patterns appear across sub-Saharan Africa and South Asia, where local sourcing, shared infrastructure and rapid reallocation of capital and labour enhance SME resilience to market and climatic shocks. ^32– 37^ Observed differences across contexts largely reflect variations in institutional capacity, market integration and regulatory environments. For instance, the ability of Ghanaian cocoa processors to shift between domestic and export markets contrasts with the more biologically constrained adjustments seen among Ugandan poultry SMEs responding to disease outbreaks. ^33, 34^ These contextual disparities underscore the need for differentiated policy interventions. Policies should strengthen access to affordable finance for sustainability-enhancing investments, support cooperative and shared-resource models, and expand entrepreneurial training and extension services to improve strategic allocation capabilities. ^21, 31, 39^ Targeted incentives for local input utilisation, investment in rural infrastructure, and risk-mitigation mechanisms such as agricultural insurance would further reinforce the positive linkage between entrepreneurial resource allocation and sustainable agribusiness SME performance in emerging economies.

### Innovation adoption, technological advancement and sustainability of agribusiness SMEs

The literature shows strong alignment on the role of entrepreneurial cognition in driving innovation adoption as a key determinant of agribusiness SME sustainability in emerging economies. ^21, 40, 41^ Empirical evidence from Vietnam, Kenya, Peru, India and Nigeria consistently indicates that opportunity-oriented entrepreneurs adopt process, product and digital innovations that enhance productivity, reduce waste and improve market access, thereby strengthening economic and environmental sustainability. ^42, 45– 51^ These findings resonate with earlier studies that link entrepreneurial orientation to higher innovation propensity and competitive advantage in resource-constrained agrarian contexts. ^40, 44, 48^ However, contradictions appear regarding the depth and inclusivity of technological advancement. While mechanisation, automation and digital tools improve efficiency, several studies underplay barriers related to cost, skills and infrastructure, which may limit adoption among smaller or more remote SMEs. Prior research highlights that innovation benefits often concentrate among firms with better access to finance, training and networks, suggesting uneven sustainability outcomes across the SME spectrum, a concern insufficiently addressed in the reviewed cases. ^41, 49^ Gaps remain in understanding long-term adoption trajectories, particularly how SMEs sustain and upgrade technologies over time, as well as the social implications of innovation, including labour displacement, gendered access to technology and digital exclusion. Further research is required to examine these dynamics through longitudinal and comparative designs across different institutional settings.

Comparative evaluation with previous studies reinforces the conclusion that sustainability-oriented and digital innovations strengthen the linkage between entrepreneurial mindset and SME performance by integrating economic viability with environmental stewardship and social value creation. ^25, 51, 52^ Similar patterns emerge across sub-Saharan Africa, South Asia and Latin America, where water-saving technologies, renewable energy solutions and mobile-based market platforms enhance resilience to climate variability and market volatility. ^25, 46, 50^ Differences observed across countries largely reflect disparities in policy support, infrastructure quality and innovation ecosystems. For example, the successful adoption of solar drying in Peru contrasts with slower diffusion of clean technologies in parts of Africa where energy access and technical support remain limited. ^46, 49^ These variations highlight the need for targeted policy interventions. Governments and development partners should prioritise innovation financing tailored to SMEs, strengthen extension and technical training services, and invest in rural digital and energy infrastructure. ^21, 48, 53^ Policy incentives that promote environmentally sustainable technologies, alongside inclusive digital platforms and innovation hubs, would further reinforce the positive impact of entrepreneurial innovation adoption on the long-term sustainability of agribusiness SMEs in emerging economies.

### Market positioning competitive advantage and sustainability of agribusiness SMEs

The literature consistently indicates that entrepreneurial cognition plays a decisive role in shaping market positioning strategies that enhance the sustainability of agribusiness SMEs in emerging economies. ^54, 55^ Evidence from Colombia, Indonesia, Morocco and the Philippines shows strong alignment with earlier studies that associate opportunity recognition, niche targeting and brand differentiation with improved economic returns and social value creation. ^56– 60, 62^ Positioning strategies centred on fair trade, organic certification, health-conscious branding and ethical sourcing reinforce competitive advantage while embedding environmental and social objectives within business models. These findings mirror prior research that highlights differentiation and value-based branding as critical mechanisms through which SMEs overcome scale disadvantages and price competition. ^58, 59, 67^ Contradictions emerge, however, regarding the durability of niche positioning under market saturation and certification costs. Some earlier studies caution that reliance on premium or ethical markets may expose SMEs to volatility in consumer preferences and compliance expenses, risks that receive limited attention in the reviewed cases. ^59, 68^ Gaps persist around how smaller or informally organised enterprises sustain branding investments over time, as well as how power asymmetries within value chains affect the distribution of gains from improved market positioning. Further research is required to examine long-term competitiveness, inclusivity and resilience of niche strategies, particularly under global demand shocks and tightening sustainability standards.

Comparative evaluation with previous studies reinforces the view that adaptive pricing and strategic value chain integration strengthen the linkage between entrepreneurial mindset and sustainable competitive advantage. ^62– 70^ Similar patterns appear across Africa, Asia and Latin America, where flexible pricing, direct buyer relationships and coordinated value chain participation improve cash flow stability, reduce transaction costs and enhance market resilience. ^63, 65, 69^ Differences across contexts largely reflect variations in market structure, institutional support and access to export channels. For instance, direct international linkages in Sri Lankan cinnamon SMEs contrast with more locally anchored pricing strategies among Egyptian vegetable farmers, shaped by differences in infrastructure and buyer power. ^64, 70^ These variations highlight the importance of supportive policy interventions. Policymakers should strengthen market access through trade facilitation, support certification and branding initiatives for SMEs, and promote inclusive value chain governance frameworks that protect smaller producers. ^54, 67^ Investments in market intelligence systems, cooperative marketing platforms and entrepreneurship training focused on strategic positioning would further enhance the capacity of agribusiness SMEs to convert entrepreneurial cognition into sustained competitive advantage and long-term sustainability in emerging economies.

### Risk management, resilience building and sustainability of agribusiness SMEs

The literature shows strong convergence on the role of entrepreneurial cognition in strengthening risk management and resilience as core drivers of agribusiness SME sustainability in emerging economies. ^71– 73^ Evidence from Zimbabwe, Cambodia, Senegal, Nepal, Peru and Vietnam consistently supports earlier studies that associate proactive risk anticipation, diversification strategies and financial buffering with improved business continuity and long-term performance under uncertainty. ^74– 82^ These findings align with prior research emphasising that entrepreneurial orientation enables SMEs to move beyond reactive coping towards anticipatory and adaptive risk management, particularly in environments characterised by climate variability, price volatility and weak institutional support. ^72, 76, 79^ However, contradictions emerge regarding the capacity of smaller and informally organised SMEs to implement sophisticated risk management tools such as early warning systems or financial reserves. Some earlier studies suggest that limited access to technology, insurance and formal finance constrains resilience-building efforts, potentially widening sustainability gaps between better-resourced and more vulnerable enterprises, an issue insufficiently addressed in the reviewed cases. ^80, 83^ Gaps remain in understanding how social capital, informal risk-sharing mechanisms and gendered decision-making influence resilience outcomes, alongside limited longitudinal evidence on the cumulative effectiveness of diversification and environmental adaptation strategies. These gaps indicate a need for further research examining resilience as a dynamic, multi-level process shaped by both entrepreneurial cognition and structural constraints.

Comparative evaluation with previous studies reinforces the argument that integrated economic, financial and environmental risk strategies strengthen the linkage between entrepreneurial mindset and sustainable SME performance. ^83– 86^ Similar patterns emerge across Africa, Asia and Latin America, where diversification, collective finance and climate-smart practices reduce vulnerability and enhance adaptive capacity. ^77, 81, 85^ Differences across contexts largely reflect variations in exposure to climate risk, institutional support and market integration. For example, climate adaptation strategies among Kenyan tea farmers and Bolivian quinoa producers reflect agro-ecological pressures distinct from the market-driven risks faced by Zimbabwean maize millers or Peruvian cacao cooperatives. ^74, 81, 85, 86^ These contextual differences underscore the importance of targeted policy interventions. Policymakers should expand access to affordable insurance, climate information services and early warning systems, while strengthening cooperative finance and extension support tailored to SME needs. ^71, 79, 84^ Investment in climate-resilient infrastructure, risk management training and inclusive financial instruments would further enable agribusiness SMEs to translate entrepreneurial cognition into resilience and sustainability across emerging economies.

### Social and environmental sustainability integration and sustainability outcomes of agribusiness SMEs

The literature demonstrates strong alignment on the role of entrepreneurial cognition in integrating social and environmental sustainability into the strategic decisions of agribusiness SMEs in emerging economies. ^87, 88^ Empirical evidence from Malawi, Indonesia, Egypt and Colombia supports earlier studies that associate long-term entrepreneurial vision with inclusive employment practices, equitable value distribution and responsible natural resource management. ^89– 93^ These findings reinforce the argument that entrepreneurs who internalise sustainability as a strategic objective embed social inclusion and ecological stewardship into core business operations, thereby strengthening operational stability and reputational capital. ^21, 91^ However, contradictions emerge regarding the costs and feasibility of sustained integration. While cooperative models, renewable energy adoption and biodiversity-friendly practices generate long-term benefits, some prior studies highlight short-term financial pressures, certification costs and compliance burdens that may deter smaller SMEs from fully adopting such practices. ^94, 95^ These challenges receive limited critical attention in the reviewed cases. Gaps remain in understanding how informal enterprises navigate sustainability integration without formal certification, how social benefits are distributed within communities, and how trade-offs between profitability and environmental protection evolve over time. Further research is required to examine these dynamics longitudinally, particularly in contexts with weak regulatory enforcement and limited access to green finance.

Comparative evaluation with previous studies confirms that regulatory alignment, responsible resource use and community-centred strategies strengthen the linkage between entrepreneurial mindset and sustainable SME performance. ^96– 102^ Similar patterns appear across Africa, Asia and Latin America, where traceability systems, organic certification and ecosystem-based production enhance market access while reducing environmental and social risks. ^97, 98, 102^ Differences across contexts largely reflect variations in institutional capacity, market incentives and ecological pressures. For example, certification-driven sustainability in Tanzanian vegetable farming contrasts with community-led conservation approaches among Moroccan argan cooperatives, shaped by differing regulatory and cultural environments. ^97, 101^ These variations highlight the need for context-sensitive policy interventions. Policymakers should reduce the cost of sustainability compliance through subsidies and technical support, strengthen cooperative governance structures, and expand access to green finance and sustainability-focused extension services. ^87, 95, 100^ Policies that reward inclusive employment, ecosystem conservation and responsible production would further enable agribusiness SMEs to translate entrepreneurial cognition into integrated social, environmental and economic sustainability within emerging economies.

### Theoretical implications

The study examines relationship between literature review and the underpinning theoretical framework thereby identifying the persistent challenges and eminent gaps as shown in Table 5.

**Table 5.** Theoretical implications and policy insights of entrepreneurial cognition on agribusiness SME sustainability in emerging economies.

Strategic domain	Theoretical implications	Policy implications
**Resource Allocation & Optimisation**	Entrepreneurial cognition guides investment prioritisation, cost management, and flexible resource deployment under scarcity; outcomes align with Triple Bottom Line through economic efficiency, environmental conservation, and social embeddedness. ^16– 39^ Structural constraints and cognitive heterogeneity limit effectiveness.	Improve access to affordable finance, support cooperative/shared infrastructure, provide capacity-building in strategic thinking, sustainability literacy, and risk-informed allocation, and incentivise water-efficient technologies, local input use, and inclusive employment. ^19, 22, 31, 35, 37, 39^
**Innovation Adoption & Technological Advancement**	Cognitive attributes (opportunity recognition, learning orientation, problem-solving heuristics) shape adoption of mechanisation, digital, and sustainability-oriented innovations; outcomes enhance economic efficiency, reduce environmental impacts, and support social welfare. ^16– 54^ Barriers include cost, skills gaps, infrastructure deficits, and risk aversion.	Provide affordable innovation financing, technology leasing, subsidies for climate-smart technologies, strengthen extension and digital literacy programs, promote innovation hubs and cooperative technology platforms, and incentivise sustainable production and renewable energy. ^16, 19, 21, 25, 40– 54^
**Market Positioning & Competitive Advantage**	Cognitive framing drives niche targeting, brand differentiation, pricing flexibility, and value chain integration; Triple Bottom Line outcomes include economic value, social inclusion, and environmentally responsible practices. ^16– 70^ Power asymmetries, certification costs, and volatile demand pose challenges.	Strengthen market intelligence, certification, and branding support; promote cooperative marketing and inclusive value chain governance; provide capacity-building in strategic marketing, pricing, and negotiation; incentivise ethical branding and traceability. ^16, 19, 54– 70^
**Risk Management & Resilience**	Entrepreneurial cognition informs proactive strategies (diversification, financial buffering, climate adaptation), enhancing continuity, social stability, and environmental protection. ^16– 86^ Structural constraints and social capital dynamics can limit effectiveness.	Expand access to insurance and emergency funds; invest in early warning systems, climate services, and technical extension; support cooperative risk-sharing, collective savings, and shared infrastructure. ^19, 20, 71– 86^
**Social & Environmental Sustainability Integration**	Long-term vision, opportunity recognition, and strategic judgement drive inclusive employment, cooperative profit-sharing, sustainable resource use, and regulatory compliance; outcomes align with Triple Bottom Line. ^16– 102^ Cost, capacity, and knowledge barriers constrain full integration.	Provide technical support, training, and extension services; incentivise renewable energy, organic certification, and community-based resource management; strengthen cooperative structures and access to green finance. ^89– 101^

### Linking entrepreneurial cognition and sustainability theories to resource allocation and optimisation

The literature on resource allocation and optimisation demonstrates strong conceptual alignment with Entrepreneurial Cognition Theory and the Triple Bottom Line framework, particularly in explaining how owner-managers in agribusiness SMEs make strategic decisions under conditions of scarcity and uncertainty. ^16– 20^ Empirical examples from Kenya, Bangladesh, Nigeria, Ethiopia, Ghana, Uganda, India and Tanzania illustrate how cognitive elements such as opportunity recognition, prioritisation heuristics and adaptive judgement guide investment choices, cost management and flexible resource deployment. ^21– 39^ These patterns directly reflect Mitchell et al.’s argument that entrepreneurial action is shaped by how information is interpreted rather than by objective conditions alone. ^16– 18^ At the same time, the outcomes described in the literature extend beyond economic efficiency to include environmental conservation and social embeddedness, consistent with the Triple Bottom Line perspective. ^19, 20^ However, persistent challenges emerge in translating these theoretical assumptions into practice. The literature tends to under-theorise structural constraints such as unequal access to finance, weak institutions and power asymmetries within value chains, which may limit the effectiveness of entrepreneurial cognition in shaping resource allocation outcomes. ^22, 28, 32^ A further gap lies in the limited interrogation of cognitive heterogeneity among entrepreneurs, as studies often assume a uniform entrepreneurial mindset despite differences in gender, education, experience and socio-cultural context. This constrains the explanatory depth of Entrepreneurial Cognition Theory when applied to diverse agribusiness settings. Limited longitudinal evidence also weakens the application of the Triple Bottom Line framework, as most studies focus on short- to medium-term efficiency gains rather than long-term trade-offs between economic survival, environmental protection and social equity.

From a policy perspective, the gaps identified between theory and practice highlight the need for interventions that strengthen the enabling environment within which entrepreneurial cognition operates. ^20, 21^ Policies should prioritise improving access to affordable and flexible finance to allow cognitively driven resource prioritisation to translate into tangible sustainability investments, particularly for smaller and marginalised agribusiness SMEs. ^22, 25^ Institutional support for cooperative models and shared infrastructure would reduce individual resource burdens and enhance collective outcomes, reinforcing both cognitive decision-making and Triple Bottom Line objectives. ^31, 35^ Targeted capacity-building programmes focused on strategic thinking, sustainability literacy and risk-informed resource allocation would also help diversify entrepreneurial cognition across different SME profiles. ^16, 17^ Environmental and social incentive schemes, including subsidies for water-efficient technologies, local input use and inclusive employment practices, would further align entrepreneurial decision-making with sustainability goals. ^19, 37, 39^ Together, such policy interventions would bridge the persistent gaps between theoretical expectations and empirical realities, strengthening the role of entrepreneurial cognition in driving resource allocation strategies that sustain agribusiness SMEs in emerging economies over time.

### Aligning innovation adoption with entrepreneurial cognition and sustainability theory

The literature on innovation adoption and technological advancement shows strong theoretical coherence with Entrepreneurial Cognition Theory and the Triple Bottom Line framework, particularly in explaining why agribusiness SMEs differ in their willingness and capacity to adopt productivity-enhancing, digital and sustainability-oriented innovations under conditions of uncertainty. ^16– 21, 40, 41^ Empirical cases from Vietnam, Kenya, Peru, India, Nigeria, South Africa and Bangladesh illustrate how cognitive attributes such as opportunity recognition, learning orientation and problem-solving heuristics shape strategic choices around mechanisation, process innovation and digital technologies. ^25, 41– 52^ These findings directly support Mitchell et al.’s assertion that entrepreneurial decisions are filtered through mental models rather than objective resource endowments alone. ^16– 18^ At the same time, the observed outcomes align with the Triple Bottom Line logic, as innovation adoption simultaneously enhances economic efficiency, reduces environmental externalities and, in some contexts, improves social welfare through food safety and market inclusion. ^19, 20^ Persistent challenges arise, however, in the limited attention given to structural and cognitive constraints that inhibit innovation diffusion. The literature tends to underplay barriers related to affordability, skills gaps, infrastructure deficits and risk aversion, which may prevent less-resourced SMEs from translating entrepreneurial cognition into sustained technological upgrading. ^41, 48, 49^ Gaps also remain in explaining how cognitive capabilities evolve over time through learning and experience, and how social norms, gender dynamics and institutional trust shape innovation decisions, limiting the explanatory reach of Entrepreneurial Cognition Theory in heterogeneous agribusiness contexts.

From a policy standpoint, the disconnect between theoretical expectations and empirical realities points to the need for interventions that strengthen both cognitive capacity and structural support systems for innovation-led sustainability. ^20, 21^ Policies should prioritise affordable innovation financing, technology leasing schemes and targeted subsidies for climate-smart and resource-efficient technologies to reduce entry barriers for smaller agribusiness SMEs. ^48, 52^ Investment in extension services and digital literacy programmes would enhance entrepreneurial learning, enabling owner-managers to better evaluate, adapt and sustain technological innovations. ^16, 17^ Innovation hubs, public–private partnerships and cooperative technology platforms could further support knowledge sharing and collective experimentation, reinforcing the cognitive mechanisms emphasised by Entrepreneurial Cognition Theory. ^18, 44^ Environmental and social incentive frameworks, including tax relief for renewable energy use and certification support for sustainable production, would strengthen alignment with Triple Bottom Line objectives. ^19, 25, 52^ Such policy interventions would narrow existing gaps between theory and practice, enabling innovation adoption to function more effectively as a strategic pathway through which entrepreneurial cognition sustains agribusiness SMEs in emerging economies.

### Theoretical alignment of market positioning strategies with entrepreneurial cognition and sustainability

The literature on market positioning and competitive advantage demonstrates strong consistency with Entrepreneurial Cognition Theory by illustrating how agribusiness SME owner-managers interpret market signals, assess risk and identify niche opportunities under uncertainty. ^16– 18, 54, 55^ Empirical evidence from Colombia, Indonesia, Morocco, the Philippines, Egypt, Guatemala, Bolivia and Sri Lanka shows that cognitive processes such as opportunity recognition, strategic judgement and adaptive learning inform decisions on niche targeting, brand differentiation, pricing flexibility and value chain integration. ^56– 70^ These findings support the theoretical assertion that strategic choices are shaped less by objective market conditions and more by how entrepreneurs cognitively frame opportunities and threats. ^17, 18^ At the same time, the sustainability outcomes associated with premium pricing, ethical branding and inclusive value chain participation align closely with the Triple Bottom Line framework, as firms generate economic value while reinforcing social inclusion and environmentally responsible practices. ^19, 20^ Persistent challenges remain, however, in the limited theoretical treatment of power asymmetries and market access constraints that may restrict the effectiveness of cognitively driven positioning strategies. The literature tends to overlook how certification costs, buyer dominance and volatile consumer preferences may undermine the long-term viability of niche markets, exposing a gap between theoretical expectations and empirical complexity. ^58, 59, 68^

From a policy perspective, bridging the gap between entrepreneurial cognition and sustainable market outcomes requires targeted institutional support that enhances both strategic capacity and market fairness. ^20, 54^ Policies should strengthen market intelligence systems, certification support and branding assistance to reduce the cost and risk of niche positioning for agribusiness SMEs. ^56, 60^ Trade facilitation measures, cooperative marketing platforms and inclusive value chain governance frameworks would help counteract power imbalances and ensure more equitable distribution of gains from market integration. ^67, 69^ Capacity-building programmes focused on strategic marketing, pricing and negotiation skills would further reinforce the cognitive mechanisms highlighted in Entrepreneurial Cognition Theory. ^16, 17^ Incentives that reward ethical branding, traceability and environmentally responsible market practices would also strengthen alignment with Triple Bottom Line objectives. ^19, 62, 70^ Such policy interventions would enhance the ability of agribusiness SMEs to translate entrepreneurial cognition into sustainable competitive advantage across diverse emerging economy contexts.

### Entrepreneurial cognition, risk management, and resilience in agribusiness SMEs: Theoretical alignment

The literature on risk management and resilience in agribusiness SMEs demonstrates clear alignment with Entrepreneurial Cognition Theory and the Triple Bottom Line framework. ^16– 20, 71, 72^ Empirical evidence from Zimbabwe, Cambodia, Senegal, Nepal, Peru, Vietnam, Kenya, and Bolivia illustrates how cognitive processes such as opportunity recognition, strategic judgement, and risk assessment guide proactive strategies for market, financial, and environmental uncertainties. ^73– 86^ These include diversification of inputs and products, financial buffering, early warning systems, and climate-adaptive practices. The findings support Mitchell et al.’s argument that strategic decisions are shaped by entrepreneurs’ mental models and heuristics rather than objective conditions alone, explaining why SMEs operating under similar constraints may adopt divergent resilience strategies. ^16– 18^ Simultaneously, the observed outcomes align with the Triple Bottom Line perspective, as risk-informed strategies enhance economic continuity, safeguard environmental resources, and promote social stability within communities. ^19, 20^ Persistent challenges exist, however, in the under-theorisation of structural constraints such as unequal access to finance, technical knowledge, and institutional support, which can limit the ability of entrepreneurial cognition to translate into effective resilience strategies. Gaps also remain in understanding the role of social capital, gendered decision-making, and informal networks in shaping adaptive responses, limiting a comprehensive application of these theories in diverse emerging economy contexts. ^79, 83^

From a policy perspective, bridging the gap between theoretical expectations and empirical realities requires interventions that strengthen both the cognitive and structural capacities of SMEs. ^20, 71^ Policies should expand access to affordable insurance, emergency funding, and risk-mitigating financial instruments to enable owner-managers to implement proactive strategies effectively. ^80, 81^ Investment in climate information services, early warning systems, and technical extension would enhance decision-making and adaptive capacity, reinforcing the cognitive mechanisms central to Entrepreneurial Cognition Theory. ^16, 17^ Supporting cooperative risk-sharing schemes, collective savings initiatives, and shared infrastructure can further reduce vulnerability while promoting social and environmental sustainability in line with Triple Bottom Line objectives. ^19, 82, 86^ Such integrated policy interventions would enable agribusiness SMEs to translate entrepreneurial cognition into robust risk management and resilience strategies, securing long-term sustainability in volatile and resource-constrained emerging economies.

### Entrepreneurial cognition and the integration of social and environmental sustainability in agribusiness SMEs

The literature on social and environmental sustainability integration demonstrates strong theoretical alignment with Entrepreneurial Cognition Theory and the Triple Bottom Line framework. ^16– 21, 87^ Evidence from Malawi, Indonesia, Egypt, Colombia, the Philippines, Tanzania, Morocco, and Bangladesh illustrates how cognitive processes such as long-term vision, opportunity recognition, and strategic judgement shape decisions that simultaneously advance economic, social, and environmental outcomes. ^88– 102^ These include inclusive employment practices, cooperative profit-sharing, sustainable resource use, and compliance with environmental and social regulations. The findings reinforce Mitchell et al.’s assertion that entrepreneurs interpret and act upon opportunities through mental models and heuristics, enabling SMEs to embed sustainability into their operational and strategic choices despite resource constraints and environmental uncertainties. ^16– 18^ Similarly, the observed outcomes align with the Triple Bottom Line framework, as firms generate financial returns while promoting social equity and ecological stewardship. ^19, 20^ Persistent challenges arise in addressing the cost, capacity, and knowledge barriers that may prevent smaller or informal SMEs from fully integrating sustainability practices. The literature also underrepresents how social and environmental benefits are distributed across communities and ecosystems, leaving gaps in understanding the equity and long-term impacts of sustainability-oriented decision-making. ^94, 95, 98^

Policy interventions are necessary to bridge these gaps and strengthen the application of entrepreneurial cognition to sustainable outcomes. ^20, 87^ Governments and development agencies should provide technical support, training, and extension services focused on sustainability-oriented decision-making, resource-efficient technologies, and inclusive business models. ^91, 92, 96^ Incentives such as subsidies for renewable energy, organic certification, and community-based resource management would reduce barriers to compliance and encourage responsible practices. ^92, 97^ Strengthening cooperative structures and promoting access to green finance would support collective action, knowledge sharing, and risk mitigation, enabling SMEs to achieve social, environmental, and economic objectives concurrently. ^89, 101^ By embedding these policy measures, emerging economies can enhance the capacity of agribusiness SMEs to translate entrepreneurial cognition into long-term sustainable performance, ensuring resilience, market competitiveness, and inclusive development.

### Practical implications

The practical implications of this narrative review are significant for policymakers, agribusiness practitioners, and development agencies seeking to enhance the sustainability of SMEs in emerging economies. First, the findings underscore that the entrepreneurial mindset—particularly the cognitive capacities of opportunity recognition, strategic judgment, and adaptive learning—plays a pivotal role in translating limited resources into tangible sustainability outcomes. Owner-managers who strategically allocate financial, human, and material resources can enhance productivity, operational efficiency, and resilience, even in volatile environments. In practice, this highlights the importance of equipping SME entrepreneurs with decision-making tools, financial literacy, and resource management skills to maximise the impact of scarce inputs while ensuring economic, social, and environmental sustainability.

Second, innovation adoption emerges as a critical pathway through which agribusiness SMEs can improve competitiveness and sustainability. The practical implication is that SMEs should prioritise mechanisation, process improvements, and digital technologies that reduce waste, enhance product quality, and expand market access. However, practical barriers such as high technology costs, infrastructure deficits, and skills shortages necessitate interventions that provide access to affordable financing, technology leasing schemes, and targeted training programmes. Development agencies and cooperatives can play a practical role in facilitating knowledge transfer, shared infrastructure, and collective experimentation platforms to ensure that technological innovations are accessible to smaller or resource-constrained SMEs.

Third, market positioning and value chain strategies offer actionable lessons for SMEs seeking long-term competitiveness. Practical implications include targeting niche markets, adopting ethical and environmentally responsible branding, and leveraging adaptive pricing and direct buyer relationships to improve cash flow stability. For practitioners, these insights suggest the need to invest in market intelligence systems, branding support, and cooperative marketing platforms to overcome scale disadvantages and ensure equitable gains across value chains.

Fourth, risk management and resilience-building practices provide practical guidance for SMEs operating in environments characterised by climate variability, market volatility, and institutional weaknesses. Proactive strategies such as input diversification, financial buffering, climate-adaptive practices, and early warning systems enhance operational continuity and long-term viability. SMEs can implement these strategies effectively when supported by policies that expand access to insurance, emergency funding, and technical extension services. Collective approaches, including cooperative savings and shared infrastructure, also provide practical mechanisms to reduce vulnerability while promoting social and environmental sustainability.

Finally, the integration of social and environmental objectives into strategic decision-making has immediate practical relevance. SMEs that embed inclusive employment, cooperative profit-sharing, sustainable resource use, and regulatory compliance into their operations can achieve the triple bottom line while enhancing reputational capital and community trust. In practice, incentives such as subsidies for renewable energy, organic certification, and community-based resource management, alongside access to green finance, can make sustainability-oriented practices feasible and scalable. For policymakers and practitioners, this indicates that fostering entrepreneurial cognition must be complemented by structural support to ensure that SMEs can simultaneously achieve economic, social, and environmental objectives.

### Directions for future for research

Based on the review, several directions for future research emerge, reflecting gaps in both theoretical understanding and empirical evidence concerning the role of entrepreneurial cognition in sustaining agribusiness SMEs in emerging economies.

First, longitudinal studies are needed to capture the long-term impacts of entrepreneurial decision-making on economic, social, and environmental sustainability. While existing literature often focuses on short- to medium-term outcomes, the durability of resource allocation strategies, innovation adoption, and market positioning over time remains underexplored. Future research should examine how SMEs maintain and adapt these strategies under evolving market conditions, climate variability, and institutional changes.

Second, there is a need to investigate heterogeneity in entrepreneurial cognition across different SME profiles. Current studies frequently assume a uniform entrepreneurial mindset, neglecting variations arising from gender, education, experience, socio-cultural background, and cognitive diversity. Understanding how these differences influence strategic decision-making and sustainability outcomes would enhance the explanatory power of Entrepreneurial Cognition Theory in agribusiness contexts.

Third, research should explore inclusivity and equity dimensions in resource allocation, innovation adoption, and sustainability integration. Evidence suggests that better-resourced SMEs often capture most benefits, leaving smaller or informal enterprises at a disadvantage. Future studies could examine how structural constraints, social norms, power relations, and informal institutions influence the distribution of economic, social, and environmental gains among stakeholders.

Fourth, the social and environmental consequences of entrepreneurial strategies require deeper investigation. While practices such as cooperative models, climate-smart agriculture, and sustainable resource management are widely reported, their effects on community livelihoods, ecosystem health, and social equity over time remain insufficiently documented. Comparative studies across different ecological, cultural, and regulatory contexts could provide valuable insights into scalability and contextual adaptability of sustainability-oriented strategies.

Fifth, the dynamics of innovation adoption and technological upgrading warrant further exploration. Future research should examine how SMEs sustain, upgrade, and diffuse technological innovations over time, including the role of learning, social networks, gendered access, and institutional trust. Understanding these mechanisms will clarify how entrepreneurial cognition interacts with structural enablers to promote long-term competitiveness and resilience.

Finally, integrated research examining the interplay of multiple strategic dimensions—resource allocation, innovation, market positioning, risk management, and sustainability integration—is needed. Such holistic studies could reveal how entrepreneurial cognition drives synergistic effects across economic, social, and environmental domains, providing a more nuanced understanding of SME sustainability pathways in emerging economies.

## Conclusion

This narrative review highlights the critical role of entrepreneurial cognition in shaping the sustainability of agribusiness SMEs in emerging economies. Across diverse contexts, owner-managers’ cognitive processes—including opportunity recognition, strategic judgement, and adaptive learning—emerge as central drivers of effective resource allocation, innovation adoption, market positioning, risk management, and social and environmental integration. Empirical evidence demonstrates that when entrepreneurs strategically deploy scarce financial, human, and material resources, adopt productivity-enhancing and sustainability-oriented innovations, position their products in niche or ethical markets, and implement proactive risk and resilience strategies, they not only improve economic performance but also advance social equity and environmental stewardship. These findings underscore the theoretical alignment of Entrepreneurial Cognition Theory and the Triple Bottom Line framework, illustrating how mental models, heuristics, and long-term strategic vision translate into multi-dimensional sustainability outcomes.

Despite these positive linkages, the review also identifies persistent challenges and gaps. Structural constraints, including limited access to finance, technical knowledge, and institutional support, can hinder the effectiveness of cognitively driven strategies, particularly for smaller and more marginalised SMEs. Similarly, the literature underrepresents cognitive heterogeneity, gendered dynamics, and equity considerations, while longitudinal evidence on the durability and cumulative impacts of sustainability-oriented decisions remains limited. These gaps highlight the complexity of translating entrepreneurial cognition into sustained SME performance and reveal opportunities for policy interventions that strengthen the enabling environment, provide targeted capacity-building, and support inclusive and responsible business practices.

The practical implications of these findings are significant. Policymakers, development agencies, and support institutions should prioritise interventions that enhance both cognitive and structural capabilities, including access to affordable finance, technical extension services, innovation platforms, and sustainability-focused incentives. By fostering an ecosystem that supports strategic decision-making, skill development, and resource-efficient operations, emerging economies can enable agribusiness SMEs to achieve long-term competitiveness, resilience, and inclusive development.

This review therefore confirms that entrepreneurial cognition serves as a critical mechanism through which agribusiness SMEs navigate uncertainty and advance sustainable outcomes in emerging economies. Integrating cognitive insights with sustainability-oriented strategies provides a holistic pathway for balancing economic viability, social responsibility, and environmental stewardship. Final remarks emphasise that advancing research, policy, and practice in this area requires a nuanced understanding of contextual variability, cognitive diversity, and structural barriers, ensuring that the potential of entrepreneurial thinking is fully realised in promoting resilient, inclusive, and environmentally responsible agribusiness sectors.

### Recommendations

Based on the findings of this review, several actionable recommendations emerge for policy and practice to enhance the sustainability of agribusiness SMEs in emerging economies. Policies should first focus on strengthening the enabling environment that allows entrepreneurial cognition to translate into practical, sustainability-oriented actions. For instance, access to affordable and flexible finance is crucial, as observed in Ghanaian cocoa processors who successfully shifted between domestic and export markets by leveraging cooperative financing and shared infrastructure. Providing targeted subsidies or low-interest loans for investments in productivity-enhancing technologies, renewable energy, and water-efficient irrigation systems would enable SMEs to optimise resource allocation while maintaining environmental stewardship, as demonstrated by Egyptian date farms adopting solar-powered water pumps and drip irrigation.

Capacity-building interventions are equally critical. Extension services, technical training, and digital literacy programmes can enhance entrepreneurs’ strategic and cognitive capabilities, enabling them to adopt innovations more effectively. Success stories include Vietnamese and Kenyan agribusiness SMEs that leveraged mechanisation and mobile-based market platforms to improve productivity and market access. Supporting cooperative models and shared infrastructure allows SMEs to pool resources, mitigate individual risk, and enhance resilience. Examples from Indonesian organic rice cooperatives highlight how collective profit-sharing and cooperative governance foster equitable social outcomes while improving operational stability.

Market-oriented policies should strengthen SMEs’ competitiveness through support for niche positioning, branding, and value chain integration. Initiatives such as trade facilitation, certification support, and cooperative marketing platforms can help reduce barriers for small producers entering premium or ethical markets. Moroccan argan oil cooperatives and Philippine cacao processors illustrate how strong branding and traceability systems can attract premium pricing, promote social inclusion, and enhance environmental sustainability. Such measures also help SMEs navigate power asymmetries and volatile consumer preferences, ensuring that benefits from niche market participation are equitably distributed.

Risk management and resilience strategies require both financial and technical support. Expanding access to affordable insurance, emergency funding, and risk-mitigating financial instruments allows SMEs to implement proactive strategies effectively. Early warning systems and climate-adaptive practices, as applied by Cambodian shrimp aquaculture enterprises and Bolivian quinoa producers, demonstrate the value of combining anticipatory decision-making with structural supports to enhance business continuity under environmental and market uncertainty.

Finally, promoting social and environmental sustainability should be an integrated aspect of policy and practice. Incentives for renewable energy adoption, organic or fair-trade certification, and ecosystem-based production can reduce compliance burdens while encouraging responsible practices. Investments in community education programs and cooperative governance, as seen in Moroccan argan oil and Malawian soybean-processing SMEs, strengthen social cohesion, build local capacity, and reinforce long-term ecological stewardship.

In summary, policy and practice should adopt a multi-pronged strategy that combines financial support, capacity-building, market facilitation, risk mitigation, and sustainability incentives. By leveraging these approaches, emerging economies can enable agribusiness SMEs to harness entrepreneurial cognition effectively, achieving operational resilience, competitive advantage, and integrated social, environmental, and economic sustainability.

### Limitations of the study

The study adopted a narrative review design, which provides flexibility in synthesising diverse forms of evidence but does not follow the rigid protocols associated with systematic reviews. This approach may introduce a degree of subjectivity in study selection, interpretation, and thematic development, despite efforts to enhance rigour through independent screening and triangulation. The absence of formal meta-analytic procedures limits the ability to quantify effect sizes or establish causal relationships across studies.

The restriction of the review to studies published between 2019 and 2025, while justified by the need to capture recent and contextually relevant developments, may have excluded earlier foundational research that could provide deeper historical insights into entrepreneurial cognition in agribusiness SMEs. Although the selected period captures critical pre- and post-pandemic dynamics, it may not fully reflect long-term trends and theoretical evolution in the field.

The study relied on selected academic databases, including Scopus, Web of Science, Google Scholar, and PubMed. While these sources ensured access to high-quality and interdisciplinary research, relevant studies indexed in other regional or specialised databases may have been overlooked. The inclusion of English-language publications only also introduces potential language bias, limiting the representation of studies from non-English-speaking regions within emerging economies.

Variations in methodological approaches, contexts, and definitions across the included studies may affect the comparability of findings. Differences in how entrepreneurial cognition, sustainability, and SME performance are conceptualised and measured across regions such as sub-Saharan Africa, South Asia, and Latin America may influence the generalisability of the conclusions.

The reliance on secondary data sources means that the study depends on the quality, scope, and reporting standards of the original studies. Any limitations or biases present in the primary studies may have been carried into the review despite efforts to assess credibility and ensure methodological transparency.

Reflexivity was maintained throughout the review process; however, the researchers’ prior knowledge and perspectives on agribusiness SMEs may still have influenced the interpretation of findings and identification of themes. While reflexive practices and audit trails were used to minimise bias, complete neutrality in narrative synthesis cannot be fully guaranteed.

## Ethics and consent

Ethics and consent were not required.

## Data Availability

This narrative review does not involve any primary data.

## References

[1] Alam MFB Tushar SR Zaman SM : Analysis of the drivers of Agriculture 4.0 implementation in the emerging economies: implications towards sustainability and food security. *Green Technol. Sustain.* 2023;1(2):100021.

[2] Reardon T Liverpool-Tasie LSO Belton B : African domestic supply booms in value chains of fruits, vegetables, and animal products fueled by spontaneous clusters of SMEs. *Appl. Econ. Perspect. Policy.* 2024;46(2):390–413.

[3] Tariq MU : Innovative business models: integrating social, economic, and environmental goals in rural manufacturing. *Rural Social Entrepreneurship Development: Network-Based Manufacturing System Model.* Hershey (PA): IGI Global Scientific Publishing;2025; pp.147–174.

[4] Ogunmoroti OE : Barriers to the sustainability of agribusiness SMEs in Nigeria: structural, financial and socio-cultural factors. *Zenodo.* 2025. [Preprint].

[5] Habibullah M Kamal A : Environmental dynamism and strategic performance in small and medium enterprises. *Journal of Energy and Environmental Policy Options.* 2024;7(3):35–42.

[6] Mandych O : Strategic management paradigm in agribusiness: a holistic approach to navigate financial uncertainty and promoting sustainable growth. Stankevych S Mandych O , editors. *Ecology, Biotechnology, Agriculture and Forestry in the 21st Century: Problems and Solutions.* Tallinn: Teadmus OÜ;2024; p.363.

[7] Darby JL Fugate BS Murray JB : The role of small and medium enterprise and family business distinctions in decision-making: insights from the farm echelon. *Decis. Sci.* 2022;53(3):578–597.

[8] Zhang QF : From sustainable agriculture to sustainable agrifood systems: a comparative review of alternative models. *Sustainability.* 2024;16(22):9675.

[9] Menbere R : *Financial management practices of small and medium businesses in Addis Ketema Sub-City [doctoral dissertation].* Addis Ababa: St. Mary’s University;2025.

[10] Mudzamiri NS : *The influence of an entrepreneurial mindset on the performance of small, medium and micro enterprises in the informal sector [doctoral dissertation].* Bloemfontein: University of the Free State;2023.

[11] Olanrewaju M : The impact of entrepreneurship education on improving small and medium-size enterprises (SMEs) performance in Kwara and Oyo States, Nigeria. 2024.

[12] Akomea SY Agyapong A Ampah G : Entrepreneurial orientation, sustainability practices and performance of small and medium enterprises: evidence from an emerging economy. *Int. J. Product. Perform. Manag.* 2023 Nov 10;72(9):2629–2653.

[13] Kruja A : Entrepreneurial orientation, synergy and firm performance in the agribusiness context: an emerging market economy perspective. *Cent. Eur. Bus. Rev.* 2020;9(1):56–75. 10.18267/j.cebr.229

[14] Sinnaiah T Adam S Mahadi B : A strategic management process: the role of decision-making style and organisational performance. *J. Work-Appl. Manag.* 2023 Apr 24;15(1):37–50. 10.1108/JWAM-10-2022-0074

[15] Salmony FU Kanbach DK : Personality trait differences across types of entrepreneurs: a systematic literature review. *Rev. Manag. Sci.* 2022 Apr;16(3):713–749. 10.1007/s11846-021-00466-9

[16] Mitchell RK Busenitz LW Lant T : Toward a theory of entrepreneurial cognition: rethinking the people side of entrepreneurship research. *Entrep. Theory Pract.* 2002;27(2):93–104.

[17] Bogataia O : Cognitive Styles in Managerial Decision-Making: Conceptual Foundations and Typological Models. *SSRN 5242248.* 2025 Apr 17.

[18] Midtgård K Selart M : Cognitive Biases in Strategic Decision-Making. *Administrative Sciences.* 2025 Jun 13;15(6):227.

[19] Elkington J : *Cannibals with forks: the triple bottom line of 21st century business.* Oxford: Capstone Publishing;1997.

[20] Nogueira E Gomes S Lopes JM : Unveiling triple bottom line's influence on business performance. *Discov. Sustain.* 2025 Jan 21;6(1):43. 10.1007/s43621-025-00804-x

[21] Ihou AF Mansingh JP : Pathways to farmers' entrepreneurship: the role of entrepreneurial mindset. *Frontiers in Sustainable Food Systems.* 2025 Jun 2;9:1584522. 10.3389/fsufs.2025.1584522

[22] Romsdahl PL : *Exploring Strategic Human Resource Management in Small Business: Priorities, Challenges, and Perceptions Among Owner-Managers.* University of Sioux Falls;2024. Doctoral dissertation.

[23] Isibor NJ Ewim CP Adaga EM : AN INCLUSIVE IMPACT INVESTING FRAMEWORK FOR SOCIAL ENTERPRISES: OPTIMIZING PROFITABILITY, SUSTAINABILITY **AND SOCIAL IMPACT.** *MULTIDISCIPLINARY JOURNAL OF MANAGEMENT AND SOCIAL SCIENCES.* 2025 Jul;5:2(1).

[24] Onyancha DM Mureithi SM Karanja N : Enhancing Resilience in Semi-Arid Smallholder Systems: Synergies Between Irrigation Practices and Organic Soil Amendments in Kenya. *Sustainability.* 2026 Jan 17;18(2):955. 10.3390/su18020955

[25] Haque MM Mahmud MN : Potential Role of Aquaculture in Advancing Sustainable Development Goals (SDGs) in Bangladesh. *Aquac. Res.* 2025;2025(1):6035730.

[26] Rossignoli CM Lozano Lazo DP Barman BK : Multi-stakeholder perception analysis of the status, characteristics, and factors affecting small-scale carp aquaculture systems in Bangladesh. *Frontiers in Sustainable Food Systems.* 2023 Jun 20;7:1121434. 10.3389/fsufs.2023.1121434

[27] Elemure IE : *Interrelationship between Lean and Green Manufacturing Practices Towards Achieving Business Performance and Promoting Sustainability: a study of manufacturing companies in Nigeria.* University of Portsmouth;2025. Doctoral dissertation.

[28] Chaudhari A : Lean, Green, and Profitable: Sustainable Operations Management Decision Frameworks. *SSRN 5103522.* 2025.

[29] Adeuyi OO : *DEVELOPMENT OF ELECTRONIC MARKET APPLICATION AND ITS POTENTIAL ADOPTION AMONG CASSAVA VALUE CHAIN ACTORS IN SOUTHWEST NIGERIA.* Abeokuta: Department of Agricultural Extension and Rural Development, College of Agricultural Management and Rural Development, Federal University of Agriculture;2024. Doctoral dissertation.

[30] Adeniyi VA : *CASSAVA PROCESSING TECHNOLOGY USAGE AND LIVELIHOOD OF WOMEN PROCESSORS IN NORTH CENTRAL NIGERIA.* Omu Aran, Kwara State): Landmark University;2022. Doctoral dissertation.

[31] Addisie G Tebarek L : Upgrading opportunities and challenges for small coffee producers in SiZighan S, Abualqumboz M, Dwaikat N, Alkalha Z. The role of entrepreneurial orientation in developing SMEs resilience capabilities throughout COVID-19. The International Journal of Entrepreneurship and Innovation. 2022 Nov;23(4):227-39.dama region of Ethiopia. *Int. J. Rural. Manag.* 2023 Aug;23(2):227–239. 10.1177/14657503211046849

[32] Hamzah MI : Market, Entrepreneurial Orientations, Crisis Adaptiveness, and Performance of SMEs: A Moderated Mediation Analysis. *SAGE Open.* 2025 Oct;15(4):21582440251390323. 10.1177/21582440251390323

[33] Jabbi E Sawitri DK : Supply Chain Resilience in the Cocoa Industry Amid Global Disruptions: A Comparative Case Study of Ghana and Côte d’Ivoire. *Proceedings of International Conference on Economics Business and Government Challenges.* 2025 Oct 28; Vol.8(1): pp.7–24.

[34] Kasima JS : Unravelling the Flock Dynamics and constraints to Poultry Production in a typical Indigenous Poultry-keeping community in Uganda. *Science and Development.* 2024 Dec 20;9(1):120.

[35] Al-Dhubaibi AA Khan AM Sharaf-Addin HH : Strategic integration: fostering sustainability in low-income country through poverty alleviation, carbon efficiency, energy and economic resilience. *Int. J. Energy Econ. Policy.* 2024;14(3):524–532.

[36] Attah RU Garba BM Gil-Ozoudeh I : Strategic partnerships for urban sustainability: Developing a conceptual framework for integrating technology in community-focused initiatives. *GSC Adv Res Rev.* 2024;21(2):409–418.

[37] Higgins A Bruce C McFallan S : Enhancing farmer linkages to markets in developing countries through mapping of supply chains and optimising transport. *Case Studies on Transport Policy.* 2023 Mar 1;11:100952. 10.1016/j.cstp.2023.100952

[38] Vats S : SUSTAINABLE AGRICULTURE PRACTICE: A REVIEW OF EMERGING POLICIES AND PRACTICES IN INDIA. *Lex Localis: Journal of Local Self-Government.* 2025 Nov 2;23:919–939. 10.52152/801883

[39] Bimbiga W Pastor D : Assessment of the Efficiency of the Dairy Supply Chain in Micro-Processing Centers in Arusha City, Tanzania. *Journal of Policy and Development Studies.* 2024 Dec 1;16(2):247–262. 10.4314/jpds.v16i2.16

[40] Zhang YZ Phiri ZP : *Sustainable Entrepreneurial Mindsets and Process: A Case Study of Smallholder Farms in Eswatini.* IUJ Research Institute, International University of Japan;2025 Mar.

[41] Terán-Yépez E Jiménez-Castillo D Sánchez-Pérez M : The effect of international opportunity recognition processes on problem-solving competence: how does past negative entrepreneurial experience matter?. *Oeconomia Copernicana.* 2022;13(2):541–579.

[42] Ngo VT : *Status and causes of rice loss in Vietnamese rice processors: a case study on Vietnamese rice processor in Mekong Delta, Vietnam: a thesis presented in partial fulfillment of the requirements of the degree of Master of Food Technology at Massey University, Manawatu, New Zealand.* Massey University;2025. Doctoral dissertation.

[43] Frare AB Beuren IM : The role of green process innovation translating green entrepreneurial orientation and proactive sustainability strategy into environmental performance. *J. Small Bus. Enterp. Dev.* 2022 Aug 9;29(5):789–806. 10.1108/JSBED-10-2021-0402

[44] Andersén J : An attention-based view on environmental management: The influence of entrepreneurial orientation, environmental sustainability orientation, and competitive intensity on green product innovation in Swedish small manufacturing firms. *Organ. Environ.* 2022 Dec;35(4):627–652. 10.1177/10860266221101345

[45] Kariuki FM : *The Effect of Value Addition Strategies on the Performance of Smallholder Dairy Farmers in Kiambu County, Kenya.* 2025. Doctoral dissertation.

[46] Becquer Frauberth CL Miguel Ángel QS Erika Amelia DL : Design of a solar and gas dryer to use coffee pulp in food processes in Peru. *Caspian Journal of Environmental Sciences.* 2023 Jul 1;21(3):509–516.

[47] Delgado-Plaza E Carrillo A Valdés H : Key processes for the energy use of biomass in rural sectors of Latin America. *Sustainability.* 2022 Dec 22;15(1):169. 10.3390/su15010169

[48] Seddaoui R Larabi C : Strategic decision-making in SMEs: examining the influence of marketing managers’ capabilities on firm performance in emerging markets through mediating and moderating mechanisms. *EuroMed. J. Bus.* 2025 Sep 22;1–35. 10.1108/EMJB-10-2024-0278

[49] Hamzat L Abiodun D Joseph A : Empowering entrepreneurial growth through data-driven financial literacy, market research, and personalized education tool. *World Journal of Advanced Research and Reviews.* 2023;19:1692–1711.

[50] Sudha S Ganeshkumar C Kokatnur SS : Adoption of mobile applications (apps) for information management in small agribusiness enterprises–an exploratory mixed-methods study of Farmer Producer Companies in India. *Global Knowledge, Memory and Communication.* 2024 Sep 20. 10.1108/GKMC-12-2023-0470

[51] Jimoh LO Kehinde AL Akintunde OK : The dynamics of postharvest loss management among cassava farmers in the Iwo ADP Zone, Osun state in Nigeria. *Journal of Agribusiness and Rural Development.* 2025;75:78–89. 10.17306/J.JARD.2025.00005R1

[52] Klerk S de Scheepers MD McIntyre K : Collective entrepreneurship and sustainable innovation: case Studies of regional Australian agribusinesses. *J. Small Bus. Entrep.* 2025 Mar 4;37(2):198–222. 10.1080/08276331.2023.2294952

[53] Ntshidi Z Dzikiti S Mazvimavi D : Effect of different irrigation systems on water use partitioning and plant water relations of apple trees growing on deep sandy soils in the Mediterranean climatic conditions, South Africa. *Sci. Hortic.* 2023 Jul 1;317:112066. 10.1016/j.scienta.2023.112066

[54] Nainggolan AF Patiung M Susilo A : Exploring the Relationship between Entrepreneurial Orientation and Performance of Agribusiness SMEs in Balikpapan. *Aurora: Journal of Emerging Business Paradigms.* 2024 Jun 30;1(1):18–29. 10.62394/aurora.v1i1.108

[55] Yuwono MA Ellitan L : Implementation of enterprise risk management as a strategy for increasing competitive advantage: Study at companies in Central Kalimantan. *Journal of Business Management and Accounting (JBMA).* 2025;15(1):55–78.

[56] Gómez SM : Market Analysis and Entry Strategy for German Investors in the Specialty Coffee Segment of the Colombian Coffee Industry. *Frontiers in Management Science.* 2024 Apr 9;3(2):8–12. 10.56397/FMS.2024.04.02

[57] Kusumawijayanti AR Sa’adah DL Sari HP : Marketing Mix Based on Local Wisdom: A Case Study of Coconut Sugar Products in Ngoran Village. *International Conference on Multidisciplinary Studies Integrating Entrepreneurial Strategies and Digital Transformation.* 2025 Oct 23; Vol.1(1): pp.727–740.

[58] Novita D : AGRIBUSINESS PRODUCT DIFFERENTIATION AND BRANDING STRATEGY TO INCREASE COMPETITIVENESS IN THE GLOBAL MARKET. *INTERNATIONAL JOURNAL OF FINANCIAL ECONOMICS.* 2025 Apr 17;2(4):49–59.

[59] Belgibayeva A Denissova O Kozlova M : Analysis of sustainable development of SMEs in agriculture. *Journal of Environmental Management & Tourism.* 2022 Jul 1;13(3):681–694. 10.14505/jemt.v13.3(59).09

[60] Montanari B Handaine M Id Bourrous J : Argan oil trade and access to benefit sharing: a matter of economic survival for rural women of the Souss Massa, Morocco. *Hum. Ecol.* 2023 Oct;51(5):995–1007. 10.1007/s10745-023-00453-6

[61] Kamiloglu S Yolci-Omeroglu P Copur OU : Making cocoa origin traceable. *Trends in sustainable chocolate production.* 2022 Feb 24;189–228. 10.1007/978-3-030-90169-1_6

[62] Basu S Munjal S Budhwar P : Entrepreneurial adaptation in emerging markets: Strategic entrepreneurial choices, adaptive capabilities and firm performance. *Br. J. Manag.* 2022 Oct;33(4):1864–1886. 10.1111/1467-8551.12572

[63] Saura JR Bužinskienė R : Behavioral economics, artificial intelligence and entrepreneurship: an updated framework for management. *Int. Entrep. Manag. J.* 2025 Dec;21(1):1–33. 10.1007/s11365-025-01076-7

[64] Mahmoud Sayed Agbo H : Forecasting agricultural price volatility of some export crops in Egypt using ARIMA/GARCH model. *Review of Economics and Political Science.* 2023 Apr 18;8(2):123–133. 10.1108/REPS-06-2022-0035

[65] Blum NW : *A Geographical Framework of Care Within Food Systems in the Kaqchikel Highlands of Guatemala.* University of Kansas;2025. Master's thesis.

[66] Carte L Schmook B Radel C : Linkages between international migration and agrarian extractivism in Guatemala. *J. Peasant Stud.* 2025 Jun 20;1–26. 10.1080/03066150.2025.2504418

[67] Anwar UA Rahayu A Wibowo LA : Supply chain integration as the implementation of strategic management in improving business performance. *Discov. Sustain.* 2025 Feb 14;6(1):101. 10.1007/s43621-025-00867-w

[68] Fontoura P Coelho A : More cooperative… more competitive? Improving competitiveness by sharing value through the supply chain. *Manag. Decis.* 2022 Feb 22;60(3):758–783. 10.1108/MD-09-2020-1225

[69] Villegas-Casaverde M Prado-Canchari A Choque-Quispe K : High Andean Association Producers of Organic Quinoa: A Sustainability Study Based on Competitiveness and Performance. *Sustainability.* 2025 Apr 27;17(9):3929. 10.3390/su17093929

[70] Kodippili SP Mapa MS : Untapped Potential of Ceylon Cinnamon (Cinnamomum verum Presl. syn. C. zeylanicum Blume) to Strengthen the Sri Lankan Economy. *Young Scientist Forum (YSF) National Science and Technology Commission Sri Lanka.* p.174.

[71] Ali I Sadiddin A Cattaneo A : Risk and resilience in agri-food supply chain SMEs in the pandemic era: a cross-country study. *Int. J. Log. Res. Appl.* 2023 Nov 2;26(11):1602–1620. 10.1080/13675567.2022.2102159

[72] Hokmabadi H Rezvani SM Matos CA de : Business resilience for small and medium enterprises and startups by digital transformation and the role of marketing capabilities—A systematic review. *Systems.* 2024 Jun 20;12(6):220.

[73] Iriani N Agustianti A Sucianti R : Understanding risk and uncertainty management: A qualitative inquiry into developing business strategies amidst global economic shifts, government policies, and market volatility. *Golden Ratio of Finance Management.* 2024 Jun 16;4(2):62–77.

[74] Madzivanzira T Mvumi BM Nazare RM : A review of appropriate mechanisation systems for sustainable traditional grain production by smallholder farmers in sub-Saharan Africa with particular reference to Zimbabwe. *Heliyon.* 2024 Sep 15;10(17):e36695. 10.1016/j.heliyon.2024.e36695 39281554 PMC11400974

[75] Kong H Tuy S Kongkroy C The inland aquaculture innovation technology development in Cambodia. 2024.

[76] Alsafadi Y Aljuhmani HY : The influence of entrepreneurial innovations in building competitive advantage: the mediating role of entrepreneurial thinking. *Kybernetes.* 2024 Nov 12;53(11):4051–4073.

[77] Kumar S Beye A Gueye F : Scoping study on building resilient groundnut and millet value chains in Senegal. 2022.

[78] McGowan AL : *Adoption determinants and economic benefits of integrated pest management for Nepali vegetable farmers.* Virginia Tech;2022. Doctoral dissertation.

[79] Landi GC Iandolo F Renzi A : Embedding sustainability in risk management: The impact of environmental, social, and governance ratings on corporate financial risk. *Corp. Soc. Responsib. Environ. Manag.* 2022 Jul;29(4):1096–1107. 10.1002/csr.2256

[80] Gładysz B Kuchta D : Sustainable metrics in project financial risk management. *Sustainability.* 2022 Nov 1;14(21):14247. 10.3390/su142114247

[81] Blanco M Mosquera LE Crisostomo D : Exploring cacao business models and agroecological transitions in Ucayali, Peru. 2024.

[82] Nguyen TT Pham CM Thai VV : How Has the Aquaculture Supply Chain’s Competitiveness Changed After the COVID-19 Pandemic in Emerging Countries? The Case of Vietnam. *Sustainability.* 2025 Feb 10;17(4):1451. 10.3390/su17041451

[83] Majlingova A Kádár TS : From Risk to Resilience: Integrating Climate Adaptation and Disaster Reduction in the Pursuit of Sustainable Development. *Sustainability.* 2025 Jun 13;17(12):5447. 10.3390/su17125447

[84] Adebayo WG : Resilience in the face of ecological challenges: Strategies for integrating environmental considerations into social policy planning in Africa. *Sustain. Dev.* 2025 Feb;33(1):203–220. 10.1002/sd.3113

[85] Cherono BM : Soil Conservation Technologies for Sustainable Crop Production in Kipkelion, Kericho County, Kenya. *European Journal of Education Studies.* 2025 Apr 29;12(5). 10.46827/ejes.v12i5.5995

[86] Jovanovic Z Stikic R Jacobsen SE : Climate Change: Challenge of Introducing Quinoa in Southeast European Agriculture. *Biology and Biotechnology of Quinoa: Super Grain for Food Security.* Singapore: Springer Singapore;2022 Jan 1; pp.345–371.

[87] Ikuemonisan ES : Challenges and strategies in Nigerian agribusiness entrepreneurship for sustainable development. *CABI Agric. Biosci.* 2024 Dec 9;5(1):115. 10.1186/s43170-024-00303-5

[88] Savaget P Ozcan P Pitsis T : Social entrepreneurs as ecosystem catalysts: The dynamics of forming and withdrawing from a self-sustaining ecosystem. *J. Manag. Stud.* 2025 Jan;62(1):246–278. 10.1111/joms.13055

[89] Gondwe A Westhuizen D van der Ottermann H : Policy options for unlocking the potential of Malawi's soybean value chain. 2024.

[90] Prasetyo T Arianti FD Jauhari S : Inclusive rice seed business: Performance and sustainability. *Open Agriculture.* 2023 Dec 6;8(1):20220236. 10.1515/opag-2022-0236

[91] Cai W Zhang J Liu F : Resource-environment-society integrated responsible production: Conceptions, measurements, implementation framework. *Renew. Sust. Energ. Rev.* 2025 Feb 1;208:115025. 10.1016/j.rser.2024.115025

[92] Huber JD : Water use efficiency in agricultural irrigation in Egypt: A qualitative study on the complexities of utilising fossil groundwater resources in the face of water scarcity. 2023.

[93] Montagnini F Fierro S del : Agroforestry systems as biodiversity Islands in productive landscapes. *Integrating Landscapes: Agroforestry for Biodiversity Conservation and Food Sovereignty.* Cham: Springer International Publishing;2024 Jun 26; pp.551–588.

[94] Chen C Huang Y Wu S : How do institutional environment and entrepreneurial cognition drive female and male entrepreneurship from a configuration perspective?. *Gender in Management: An International Journal.* 2023 May 22;38(5):653–668. 10.1108/GM-04-2022-0124

[95] Tian X Zhao C Ge X : Entrepreneurial traits, relational capital, and social enterprise performance: Regulatory effects of cognitive legitimacy. *Sustainability.* 2022 Mar 12;14(6):3336. 10.3390/su14063336

[96] Bariuan K : Scalable and Sustainable Shrimp Farming Strategies for SMEs in the Philippines. 2025.

[97] Sarantila SS : Incentivising Sustainability: Institutional barriers to the adoption and sustainability of organic agriculture in Morogoro, Tanzania. 2025.

[98] Ahmad SA Teo PC : The Implementation of Enterprise Risk Management (ERM) Frameworks in Small and Medium Enterprises (SMES): A Literature Review. *International Journal of Academic Research in Business and Social Sciences.* 2024;14(9):290–307.

[99] Alkhodary D : Integrating sustainability into strategic management: A path towards long-term business success. *Int. J. Prof. Bus. Rev.* 2023;8(4):e01627. 10.26668/businessreview/2023.v8i4.1627

[100] Bhutto SM : Sustainability in Business Management: Strategies for Long-Term Success. *Journal for Business Research Review.* 2024 Mar 31;2(1):39–50.

[101] Montanari B Handaine M Id Bourrous J : Argan oil trade and access to benefit sharing: a matter of economic survival for rural women of the Souss Massa, Morocco. *Human Ecology.* 2023 Oct;51(5):995–1007. 10.1007/s10745-023-00453-6

[102] Alam MI Rahman MS Ahmed MU : Mangrove forest conservation vs shrimp production: Uncovering a sustainable co-management model and policy solution for mangrove greenbelt development in coastal Bangladesh. *Forest Policy Econ.* 2022 Nov 1;144:102824. 10.1016/j.forpol.2022.102824

