# Supplementary material for: Strategic Decisions and the Entrepreneurial Mindset in Sustaining Agribusiness SMEs in Emerging Economies: Unlocking Pathways to Resilience – A Narrative Review
Source: F1000Res. 2026 May 4;15:277. Originally published 2026 Feb 16. [Version 2] doi: 10.12688/f1000research.177782.2 (PMC13187714; doi:10.12688/f1000research.177782.2)
Supplement: Peer review history

# Reviewer report: Reviewer response for version 2

**DOI:** 10.5256/f1000research.198975.r483855

**Published:** 2026-06-10

**Authors:** Monica Kagorora Kansiime

**Affiliations:** 1 Centre for Agriculture and Bioscience International, Nairobi, Kenya

1. The author describes a narrative review but employs systematic review elements such as dual independent screening, extraction, audit trails, and explicit inclusion/exclusion criteria. The justification for mixing approaches is not fully articulated. 

 2. The manuscript states that the initial search yielded over 1,200 articles but does not specify how many were retained after screening. Could you provide a simple count and a brief justification for the most influential 20–30 studies that shaped your core findings?

 3. Across sections (Resource allocation, Innovation adoption), evidence is presented as sequential country examples (Kenya, Bangladesh, Nigeria, Ethiopia, etc.) rather than integrated conceptual themes. The reader learns what has been studied but not why these patterns emerge and how cognition specifically operates across contexts. 

 4. The author justifies the 2019–2025 timeframe as capturing pre-pandemic baseline and post-pandemic transformations. However, with only 2019 as pre-pandemic and a small number of 2020 studies, the review cannot meaningfully contrast the two periods. Also, there is no analysis comparing findings from 2019–2020 versus 2021–2025.

 5. Theoretical framework: the statement that Entrepreneurial Cognition Theory explains "why agribusiness SMEs facing similar constraints may adopt divergent strategies" is not supported by the narrative review method used here. Without comparative evidence from firms with different cognitive orientations under identical constraints, the review can describe patterns but cannot explain divergence. Please rephrase to align with the methodological limits of a narrative synthesis.

 6. The table states “2020–2025” but the text says “2019–2025”. Harmonise.

 7. Table 3: The row “Social and environmental sustainability integration” is duplicated. Delete the second occurrence. 

 8. Just curious, did the authors encounter any studies showing negative or null effects of the entrepreneurial mindset on sustainability? If so, how were they handled?

 9. The review emphasises cognitive mechanisms, but strategic decisions are also shaped by structural factors such as finance, infrastructure, and institutions. How can we confidently conclude that the observed sustainability outcomes are primarily cognitive-driven rather than resource-driven? May be helpful to include a mention of these variables too.

Is the review written in accessible language?

Yes

Are all factual statements correct and adequately supported by citations?

Partly

Are the conclusions drawn appropriate in the context of the current research literature?

Partly

Is the topic of the review discussed comprehensively in the context of the current literature?

Yes

Reviewer Expertise:

Agrifood systems policy, entrepreneurship, food security, development extension, seed systems, market systems and value chains

I confirm that I have read this submission and believe that I have an appropriate level of expertise to confirm that it is of an acceptable scientific standard, however I have significant reservations, as outlined above.

# Reviewer report: Reviewer response for version 2

**DOI:** 10.5256/f1000research.198975.r483848

**Published:** 2026-05-19

**Authors:** Asael Islas Moreno

**Affiliations:** 1 Instituto de Ciencias Agropecuarias, Universidad Autónoma del Estado de Hidalgo, Tulancingo, Mexico

After a thorough and rigorous reading of the manuscript, my recommendation is that it be approved for indexing. I find that the study presents a robust theoretical model in which entrepreneurial cognition (through its different dimensions) influences strategic decision-making (five distinct types are assessed), ultimately leading to outcomes encompassing the three pillars of sustainability (economic, environmental and social) within the context of small and medium-sized agribusiness enterprises. The model is empirically supported by the literature reviewed and, beyond providing a comprehensive state-of-the-art overview, it also develops a future research agenda and offers implications of practical and public policy relevance. I would only like to highlight a couple of concerns that remain after my full review of the manuscript.

 1. In my view, the study does not fully achieve its aim of providing a pre- and post-pandemic examination, as it only analyses publications from 2019 to 2025. In particular, I am not convinced that the pre-pandemic stage is adequately represented solely through studies from 2019 and a limited number from 2020. If the central objective was indeed to contrast the periods before and after the pandemic, a ten-year timeframe (2015–2025) would appear more appropriate.

 2. To strengthen the theoretical underpinning of the study, I believe it would be pertinent to incorporate theory specifically associated with strategic decision-making. I would therefore recommend reviewing and incorporating Olson et al. (2007), Strategic Decision Making: The Effects of Cognitive Diversity, Conflict, and Trust on Decision Outcomes.

Is the review written in accessible language?

Yes

Are all factual statements correct and adequately supported by citations?

Partly

Are the conclusions drawn appropriate in the context of the current research literature?

Yes

Is the topic of the review discussed comprehensively in the context of the current literature?

Yes

Reviewer Expertise:

Agricultural Entrepreneusrhip and Agribusiness

I confirm that I have read this submission and believe that I have an appropriate level of expertise to confirm that it is of an acceptable scientific standard.

# Reviewer report: Reviewer response for version 2

**DOI:** 10.5256/f1000research.198975.r481408

**Published:** 2026-05-14

**Authors:** Kingsley Kofi Arthur

**Affiliations:** 1 University of Energy and Natural Resources, Sunyani, Ghana

Dear Author,

 Thank you very much for the significant changes made to the main body of the manuscript following the earlier suggestions to improve its quality.

 Here are a few corrections you have to consider:

 1. The methodology will still need a few touches to sweep out some redundancies, for instance, what is the purpose of the reflexivity and data analysis in narrative review?? I suggest these sections be removed.

 2. The methodology is overly lengthy and has to be revised to make it more brief and concise. This is a narrative review and not a systematic review, as the current revised section indicates.

 3. The data collection method is repetitive. Kindly cross-check to address these methodological issues.

 4. I suggest you insert some Figures and Conceptual Frameworks in a way to demonstrate the relationships between the constructs and summarize the findings.

Is the review written in accessible language?

Yes

Are all factual statements correct and adequately supported by citations?

Partly

Are the conclusions drawn appropriate in the context of the current research literature?

Yes

Is the topic of the review discussed comprehensively in the context of the current literature?

Yes

Reviewer Expertise:

Agribusiness Management, Development Economics, Development Studies, Supply Chain Management, Economic Policy, Social Policy, Modern Slavery, Economic Criminology

I confirm that I have read this submission and believe that I have an appropriate level of expertise to confirm that it is of an acceptable scientific standard, however I have significant reservations, as outlined above.

# Reviewer report: Reviewer response for version 1

**DOI:** 10.5256/f1000research.196069.r460087

**Published:** 2026-03-30

**Authors:** Manoj P Samuel, Razia Muhammed

**Affiliations:** 1 Central Institute of Fisheries Technology, Kochi, Kerala, India

**Comments on the manuscript ‘Strategic Decisions and the Entrepreneurial Mindset in Sustaining Agribusiness SMEs in Emerging Economies’**

**1. Number of Articles and Selection Process**

 The manuscript states that the initial search yielded more than 1,200 records, but the final number of studies included after screening is not clearly specified. Although the screening criteria are described in the *Materials and Methods* section, stating the final sample size would improve methodological transparency.

**2. Coverage of Relevant Literature**

 The manuscript includes good examples from different agribusiness contexts. However, the literature base could be strengthened by incorporating additional references from specialised agribusiness and agricultural management journals, particularly those addressing SME constraints, agribusiness strategy, and sustainability transitions in agricultural value chains.

**3. Analytical Depth of the Review**

 The manuscript provides many illustrative examples from different countries. While informative, some sections read more like sequential case descriptions rather than an integrated synthesis. For example, in the section “Resource allocation and optimisation” (Pages 6–7), examples from Kenya, Bangladesh, Nigeria, Ethiopia, Ghana, and Uganda are presented consecutively. Grouping such examples into broader conceptual themes or patterns would help highlight the key mechanisms through which entrepreneurial cognition contributes to SME sustainability. Use of figures/charts is also recommended.

**4. Integration of Theoretical Frameworks**

 The study appropriately draws on Entrepreneurial Cognition Theory and the Triple Bottom Line (TBL) Framework (Pages 5–6). However, the conceptual linkage between the two frameworks could be articulated more clearly. In particular, the manuscript could elaborate on how entrepreneurial cognitive processes translate into strategic decisions that generate economic, environmental, and social sustainability outcomes. Strengthening this connection would enhance the theoretical coherence of the paper. *For example, entrepreneurs with a resilient and opportunity-oriented mindset may look beyond immediate financial gains and make decisions that also consider environmental stewardship and community well-being as part of long-term business sustainability.*

**5. Regional Nuance in the Discussion**

 The manuscript frequently refers to “emerging economies” as a broad category. However, agribusiness SMEs operate under different institutional and market conditions across regions. Differentiating between regional contexts, for example, sub-Saharan Africa, South Asia, and Southeast Asia, could add analytical depth and help explain how entrepreneurial decision-making interacts with region-specific constraints and opportunities.

**6. Consideration of the COVID-19 Context**

 The literature reviewed spans 2020–2025, overlapping with the COVID-19 pandemic and recovery period. While the paper discusses resilience and strategic decision-making, it does not explicitly examine whether the pandemic influenced entrepreneurial responses, the adoption of innovation, or risk management strategies among agribusiness SMEs. A brief reflection on this context could enhance the review's relevance. The author could also have given more emphasis on the application of circular economy concept in SMEs.

**7. Author Affiliation:** Under the author’s name, the word **"bushesnyi"** appears before the university name. Please check if this is a typo or if it was intended to be "Bushenyi."

**8. Duplication: **In Table 3, the row “Social and environmental sustainability integration” appears twice consecutively. This is likely a formatting or editing error and should be corrected by removing the duplicate row.

**9. Section Heading: **On page 18, the heading currently reads “Directions for future **for** research,” which appears to be a typographical error. It should be corrected to “Directions for Future Research.”

**10. Grammatical Clarity: **The paper is generally well written. However it seems more narrative than being concise while drawing inferences and conclusions.

****On Page 18, Future Research Section, A sentence begins with “ *research should explore inclusivity and equity dimensions in resource allocation…*”. For grammatical clarity, it would be better revised as “ *Future research should explore inclusivity and equity dimensions in resource allocation…*”.

**11. Alignment and writing style: **In many places, the article resembles a thesis report or book chapter including while stating the objectives, screening inclusion/exclusion criteria. Rather It shall be modified into the format of a research paper. The sub heading like literature review etc shall be avoided.

**Overall Evaluation**

 The manuscript addresses an important topic and provides a broad synthesis of literature on entrepreneurial cognition and sustainability in agribusiness SMEs. With clearer reporting of the study selection process, stronger analytical synthesis, and a more explicit integration of theoretical perspectives, the manuscript could make a valuable contribution to the literature on agribusiness entrepreneurship and sustainability in emerging economies. However, the manuscript looks extremely narrative, exhaustive and at times repetitive, and lacks readability. Hence the author shall try to make inferences, suggestions and conclusions in a simpler fashion, may be with the support of a diagram. The sections shall be categorised in a more systematic manner to ensure continuity, understanding and smooth read.

 The manuscript can be accepted for indexing with minor revisions.

Is the review written in accessible language?

Yes

Are all factual statements correct and adequately supported by citations?

Partly

Are the conclusions drawn appropriate in the context of the current research literature?

Yes

Is the topic of the review discussed comprehensively in the context of the current literature?

Yes

Reviewer Expertise:

IPR and technology managment, Technology transfer and commercialization, fishery engineering, water resources enegineering

We confirm that we have read this submission and believe that we have an appropriate level of expertise to confirm that it is of an acceptable scientific standard.

# Reviewer report: Reviewer response for version 1

**DOI:** 10.5256/f1000research.196069.r460079

**Published:** 2026-03-17

**Authors:** Kingsley Kofi Arthur

**Affiliations:** 1 University of Energy and Natural Resources, Sunyani, Ghana

**General Remark:**

 This study adopts the narrative review approach to examine how strategic decisions shaped by the entrepreneurial mindset sustain agribusiness SMEs in emerging economies, highlighting pathways to resilience. Drawing on documents published between 2020 and 2025, the authors found that entrepreneurial cognition guides strategic resource allocation, innovation adoption, market positioning, risk management, and the integration of social and environmental sustainability, collectively enhancing economic, social, and ecological resilience. Again, they also concluded that challenges include structural constraints, unequal access to finance and technology, gender disparities, and sustainability trade-offs in niche markets. Policy and practical implications emphasise cooperative models, targeted innovation financing, market intelligence support, and incentives for sustainability-aligned practices.

 Despite the significance of this study in contributing to some of the key factors that influence entrepreneurs' decision-making within the agribusiness industry, the study is characterised by many limitations that need to be addressed to meet indexing consideration in this journal.

 #Below are my suggestions to improve the paper's quality:

1. The authors opined that scholars have paid less attention to the cognitive foundation that links entrepreneurial thinking to strategic decision-making yet failed to provide relevant citations to back their claims. The authors must cite the relevant literature on the current subject to support this claim.
2. I am surprised the authors provided all these claims in the problem statement section yet failed to provide relevant citations to back the claims. This is science, and every sentence or statement must be backed by relevant literature to allow for the authentication of the information.
3. Under the purpose of the study, the authors must be specific, apart from the contribution of the study to the extant literature, how will the findings also benefit entrepreneurs, specifically in an emerging economy?
4. Again, I suggest that the subsections: problem statement, purpose of the study and research objectives must be carefully merged with the introduction instead of separating them.
5. In the introduction section of the paper, the authors highlighted that they adopted a narrative review approach; however, the methodology reflects that of a systematic review, as indicated by the authors. Narrative review and systematic literature review are some of the types of review studies that employ different research designs.
6. I suggest the authors adopt a comprehensive systematic literature review technique following a predefined protocol to help achieve the objectives of the study (clue: get more abreast with the latest PRISMA protocol).
7. Again, in the methodology section, for each search string or key term, the authors must indicate the number of documents retrieved while demonstrating how these results were merged and subjected to the screening criteria.
8. What informed the limitation on the year range (2019-25) and the selection of the specific databases? A brief justification should be provided to justify these selections.
9. A PRISMA framework must also be included to indicate how the documents were identified and retrieved from the various databases.
10. Insert a table to show how the key terms/algorithms were combined to search for the documents from the various academic databases indicated by the study. This can strengthen the reproducibility of the study.
11. Contradiction: In the data collection methods, the authors indicate that the publication year range was from 2019-2020(see page 4, third line), however, under the screening and inclusion section, the publication year indicated 2020-2025. This has to be corrected.
12. How was the study quality and assessment performed (risk of bias)? This is very important and has to be indicated to increase the validity and credibility of the study.
13. In a systematic review, assessing paper quality based on the impact factor of the journal can increase the risk of selection bias. Documents included in the study should only be based on the quality of the study and how they can help the researcher achieve the objective of the study.
14. The methodology section is entirely unclear, and difficult to understand the technique adopted in finding the pertinent papers to achieve the objectives of the study. I will suggest that the authors take their time and read the recent systematic review study by Arthur et al. (2026 to structure the methodological section.

(refer to 1,2)

1. There is nothing like a literature review in a systematic review; therefore, the literature review section of the paper should be changed to Emergent themes. Articles included should also be cited under their relevant theme.
2. How were the themes generated? Did the authors then derive the themes in consultation with the included literature?
3. What do the authors mean by “discussion of literature”? In fact, all the themes presented under this section should rather be presented in the new section “emergent themes”. Again, some of the claims under the themes are repetitive, making some of the academic arguments redundant.
4. Usually, the discussion section should carry a descriptive analysis on the characteristics of the documents included, as well as the annual productivity and output emanating from various sources and countries included in the study. Again, Figures and tables are encouraged to demonstrate the specific countries where these studies are coming from and what specific subjects they are focusing on. This gives a detailed perspective on how the subject has evolved and where future scholars should consider.
5. Some of the academic claims are overly lengthy, making some of the arguments convoluted. I suggest the authors summarise some sections of the paper while keeping to the relevant points consistent with the objective and research questions underpinning the study.
6. Conclusions should first be drawn before proceeding to offer implications for practices and future research.
7. How different is the recommendation from the implications for practices and future research?? They literally serve the same purpose; the authors should carefully merge and trim down the excessive information in these sections.
8. The study is poorly formatted with no proper labelling of the various sections.
9. The predefined protocol used by the authors should be inserted in the manuscript or supplied as an appendix.
10. How does this review advance theory within the entrepreneurship domain? This section is very important and has to be highlighted to demonstrate its relevance in contributing to theory.
11. Limitations of the study have to be acknowledged.
12. Some of the references have incorrect issue, volume and page numbers.
13. Some of the in-text citations are missing in the references list **.**

Is the review written in accessible language?

Yes

Are all factual statements correct and adequately supported by citations?

Partly

Are the conclusions drawn appropriate in the context of the current research literature?

Yes

Is the topic of the review discussed comprehensively in the context of the current literature?

Yes

Reviewer Expertise:

Agribusiness Management, Development Economics, Development Studies, Supply Chain Management, Economic Policy, Social Policy, Modern Slavery, Economic Criminology

I confirm that I have read this submission and believe that I have an appropriate level of expertise to confirm that it is of an acceptable scientific standard, however I have significant reservations, as outlined above.

## References

[1] : The art of writing literature review: What do we know and what do we need to know?. *International Business Review* .2020;29(4) : 10.1016/j.ibusrev.2020.101717 10.1016/j.ibusrev.2020.101717

[2] : Literature reviews as independent studies: guidelines for academic practice. *Review of Managerial Science* .2022;16(8) : 10.1007/s11846-022-00588-8 2577-2595 10.1007/s11846-022-00588-8
